# The role of dopamine and endocannabinoid systems in prefrontal cortex development: Adolescence as a critical period

**DOI:** 10.3389/fncir.2022.939235

**Published:** 2022-11-01

**Authors:** Kate Zara Peters, Fabien Naneix

**Affiliations:** ^1^Sussex Neuroscience, School of Psychology, University of Sussex, Falmer, United Kingdom; ^2^The Rowett Institute, University of Aberdeen, Aberdeen, United Kingdom

**Keywords:** prefrontal cortex (PFC), adolescence, dopamine, endocannabinoid, reward, neuromodulators

## Abstract

The prefrontal cortex plays a central role in the control of complex cognitive processes including action control and decision making. It also shows a specific pattern of delayed maturation related to unique behavioral changes during adolescence and allows the development of adult cognitive processes. The adolescent brain is extremely plastic and critically vulnerable to external insults. Related to this vulnerability, adolescence is also associated with the emergence of numerous neuropsychiatric disorders involving alterations of prefrontal functions. Within prefrontal microcircuits, the dopamine and the endocannabinoid systems have widespread effects on adolescent-specific ontogenetic processes. In this review, we highlight recent advances in our understanding of the maturation of the dopamine system and the endocannabinoid system in the prefrontal cortex during adolescence. We discuss how they interact with GABA and glutamate neurons to modulate prefrontal circuits and how they can be altered by different environmental events leading to long-term neurobiological and behavioral changes at adulthood. Finally, we aim to identify several future research directions to help highlight gaps in our current knowledge on the maturation of these microcircuits.

## Introduction

Successful adaptation to a changing environment requires a complex set of cognitive processes known as executive functions including working memory, attentional processes, behavioral flexibility and decision-making (Rangel et al., 2008). The prefrontal cortex (PFC) plays a key role in regulating these processes by integrating multiple signals and controlling the activity of subcortical circuits (Fuster, 2001; Miller and Cohen, 2001). Altered prefrontal functioning is associated with numerous pathological states including substance use disorders, schizophrenia, eating disorders and obsessive compulsive disorders (Paulus, 2007).

In primates including humans the PFC has been historically defined as the part of the cortex that receives projections from the mediodorsal thalamic nucleus (Rose and Woolsey, 1948). In other mammals, especially rodents, the existence of the PFC and its homology to primate PFC are still debated but it is typically defined as the medial prefrontal cortex (mPFC) and the orbitofrontal cortex (see Uylings et al., 2003; Carlén, 2017; Laubach et al., 2018; Kolk and Rakic, 2022 for a complete review). Prefrontal functions are supported by local microcircuits involving long-range glutamatergic pyramidal neurons and diverse populations of GABAergic interneurons (Seamans and Yang, 2004; Lewis and González-Burgos, 2008). Importantly, prefrontal functions are highly dependent on the influence of neuromodulatory inputs (dopamine, serotonin, acetylcholine, and noradrenaline; Hoover and Vertes, 2007) as well as local endocannabinoid signaling (McLaughlin et al., 2014).

Adolescence is an important developmental period between childhood and adulthood characterized by major behavioral and cognitive changes including increased social interactions, novelty-seeking and risk-taking (Spear, 2000; Ernst et al., 2006; Casey et al., 2008; Walker et al., 2017). At the neurobiological level, adolescence is associated with neurophysiological changes, an increase in white matter volume and inverted U-shaped changes in gray matter volume related to synaptic overproduction and subsequent pruning (Lenroot and Giedd, 2006; Caballero et al., 2016; Drzewiecki and Juraska, 2020; Kolk and Rakic, 2022). These changes are especially marked in the PFC, which may participate to the development of adult-like independent behavior (Ernst et al., 2006; Somerville and Casey, 2010). However, adolescence is also associated with the onset of several major neuropsychiatric disorders (Paus et al., 2008) related with prefrontal dysfunction suggesting that prefrontal circuits may be especially vulnerable to external insults during this stage. Thus, there is a growing interest in gaining a better understanding of the maturation processes of prefrontal microcircuits and how their perturbation may lead to long-lasting neurobiological and cognitive alterations.

In this review, we focus on two important elements of prefrontal circuits: the mesocortical dopamine system and the endocannabinoid system. We outline the unique developmental profiles of these systems during adolescence, how they play essential roles in prefrontal physiology, how they interact to control prefrontal functions and how their alteration can lead to long-term changes at adulthood. Finally, we identify future research directions to highlight gaps in our current knowledge on the maturation of these microcircuits.

## Adolescence in humans and rodent models

**Figure 1:**
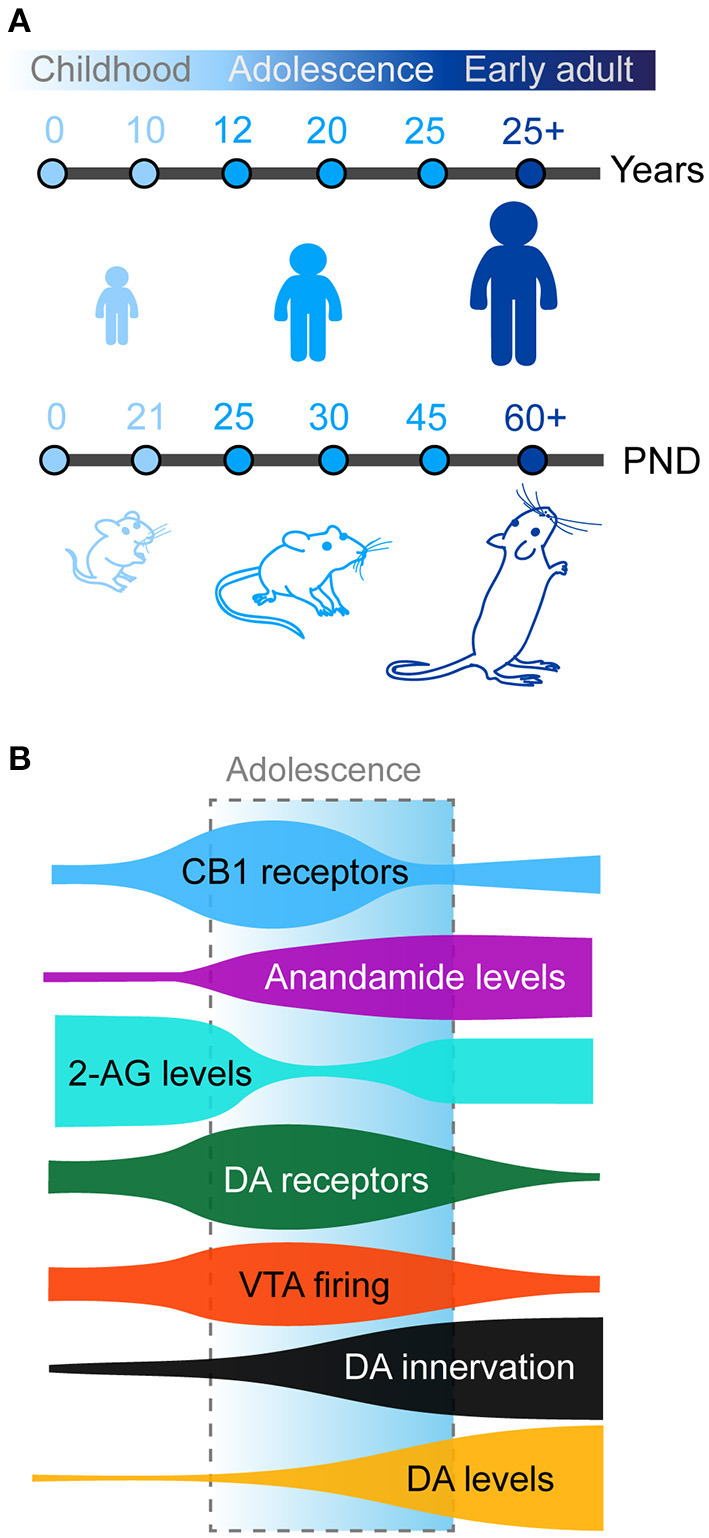
**(A)** Timing of adolescence in humans (top) and rodents (bottom). Adolescence and peri-adolescence are defined as beginning around the age 10 years and continuing until the mid-20's in humans and from post-natal day (PND) 21 to PND 50-60 in mice and rats. **(B)** Maturation of prefrontal dopamine (DA) and endocannabinoid systems across development. Schematic shows density of prefrontal anandamide (purple), dopamine (DA) tissue concentration (orange), and DA fibers (black) steadily increase from childhood to adulthood. Meanwhile, both CB1 (blue) and dopamine receptors (green) present a transient peak of expression in the prefrontal cortex during early adolescence, paralleled with similar changes in the activity of dopamine neurons in the VTA (red). Finally 2-AG levels (cyan) transiently decreases compared to childhood before returning to stable adult levels.

Adolescence as previously defined is observed in humans but also conserved in other mammals, including rodents (mice and rats) which are commonly used as animal models in neuroscience research (Figure 1A). The exact boundaries of adolescence remain difficult to define. In humans, this is commonly defined as spanning between 12 and 18 years of age. However, these boundaries are not concrete and have been expanded in some studies which report behavioral changes from as early as the age of 10 with the end of adolescent brain maturation occurring as late as the mid-20's (Steinberg, 2005; Ernst et al., 2006; Casey et al., 2008; Blakemore, 2012; Blakemore and Robbins, 2012). Neurocognitive and neurobiological comparisons between primates and rodents have been a somewhat controversial topic in recent years. In one of the most influential reviews on this topic, Spear initially defined adolescence in rodents between post-natal day (PND) 28 and 42 based on the neurobehavioral characteristics reported, however this paper highlighted this may be too restricted (Spear, 2000). Later authors have used diverse age windows to define adolescence and peri-adolescence, based on additional behavioral and neurobiological criteria. Some authors consider rodent adolescence to begin immediately after weaning on PND21 (Schneider, 2008, 2013; Yuan et al., 2015) and to extend all the way until adulthood (PND60-70; Andersen, 2003; McCutcheon and Marinelli, 2009; Brenhouse and Andersen, 2011; Walker et al., 2017). More recently and based on changes in prefrontal anatomy, Caballero and Tseng have classified adolescent periods of development and vulnerability into early (PND35-40), mid (PND40-50), and late (PND50-60) adolescence (Caballero et al., 2016). In the rest of the manuscript, we will use the term adolescence to refer to the broad period between weaning (PND21) and adulthood (PND60-70) in rodents.

An important point to note is that although puberty is a part of adolescence, it does not itself fully define adolescence. Puberty is defined as sexual maturity resulting from changes in the secretion of gonadal hormones. In humans the onset of puberty (~10 years old) is often considered as the onset of adolescence, but this can vary depending on sex, gender, ethnicity and socioeconomical status. In rodents, these two phenomena are clearly distinct between sexes. In females, vaginal opening and first ovulation occur early during adolescence (PND30-40), whereas in males increased testosterone and preputial separation occur during mid adolescence (PND40-45; Schneider, 2008; Delevich et al., 2021). Puberty likely contributes to the development of prefrontal anatomy and functions which we will briefly discuss later.

## The mesocortical dopamine pathway: A uniquely late maturing system

The dopamine system is a central actor in the modulation of behavioral control and conveys various signals related to prediction error, motivation and event salience (Floresco and Magyar, 2006; Berridge, 2007; Schultz, 2007; Wickens et al., 2007; Bromberg-Martin et al., 2010; Berke, 2018). It presents a uniquely late development during postnatal life especially within prefrontal regions, which appears to be fundamental for normal cognitive development (Spear, 2000; Ernst et al., 2006; Somerville and Casey, 2010; Sturman and Moghaddam, 2011; Walker et al., 2017).

### The mesocortical dopamine system

Dopaminergic cells mainly located in the midbrain differentially innervate the dorsal striatum (nigrostriatal pathway), the nucleus accumbens (mesolimbic pathway), and prefrontal regions (mesocortical pathway) which we will focus on in the present review (for recent reviews on the anatomical organization of dopamine pathways see Björklund and Dunnett, 2007; Lammel et al., 2014; Morales and Margolis, 2017). The mesocortical pathway originates from the medial part of the ventral tegmental area (VTA) and is comprised of dopamine neurons (30–40%) but also an important fraction of GABAergic and glutamate neurons (Morales and Margolis, 2017). In primates, dopamine neurons innervate prefrontal and sensorimotor cortical regions whereas in rodents this is limited to the mPFC, the agranular insular and the perirhinal cortices (Berger et al., 1991). Dopamine innervation is stronger in ventral parts of the mPFC (prelimbic cortex or Brodmann area 32 / infralimbic cortex or Brodmann area 25; Laubach et al., 2018), and is especially observed in deep cortical layers (layers V–VI; Van Eden et al., 1987; Heidbreder and Groenewegen, 2003). In contrast to the mPFC, rodent orbitofrontal regions are weakly innervated by dopamine neurons. Dopamine neurons rarely send collateral projections to other regions, meaning that mesocortical dopamine neurons represent a distinct population compared to mesolimbic neurons (Fallon, 1988; Beier et al., 2015). Dopamine neurons display two characteristic activity patterns: (1) a “tonic” low frequency mode (2–5 Hz) maintaining basal levels of dopamine in downstream targets and (2) a “phasic” mode with short high frequency (10–20 Hz) bursts transiently increasing dopamine release (Grace and Bunney, 1984a,b; Floresco et al., 2003; Lapish et al., 2007; Rice et al., 2011). This burst firing is especially observed in response to relevant environmental stimuli (Schultz, 2007; Bromberg-Martin et al., 2010). The exact functions of these different modes of dopamine signaling are still debated but through its actions at downstream targets and within local microcircuits, dopamine guides, invigorates and updates complex behaviors (Niv, 2007; Schultz, 2007; Bromberg-Martin et al., 2010; Salamone and Correa, 2012; Berke, 2018).

Mesocortical dopamine neurons respond to both appetitive (Hernandez and Hoebel, 1990; Ahn and Phillips, 1999; Bassareo et al., 2002; St Onge et al., 2012; Ellwood et al., 2017) and aversive stimuli (Abercrombie et al., 1989; Mantz et al., 1989; Vander Weele et al., 2018). Moreover, they have unique properties compared to other dopamine projections including low levels of dopamine transporter DAT and D2 autoreceptors associated with higher basal frequency (Sesack et al., 1998; Lammel et al., 2008). These properties result in different kinetics of dopamine signaling compared to subcortical regions (Garris and Wightman, 1994; Cass and Gerhardt, 1995; Vander Weele et al., 2018). Prefrontal neurons also directly regulate other dopamine circuits by exciting VTA mesocortical and mesolimbic neurons (Carr and Sesack, 2000; Heidbreder and Groenewegen, 2003; Gabbott et al., 2005; Beier et al., 2015) and by modulating dopamine release within subcortical regions including the nucleus accumbens (Taber et al., 1995; Karreman and Moghaddam, 1996; Rice et al., 2011; Nolan et al., 2020).

With its unique characteristics, the mesocortical dopamine pathway plays a key role in prefrontal function (Fuster, 2001; Miller and Cohen, 2001). Current theories postulate a “dual-state” function of prefrontal dopamine signaling. Tonic dopamine levels may act to maintain goal representation and adapted behavioral strategies by stabilizing the activity of specific prefrontal neuronal ensembles. Phasic dopamine release occurs in response to relevant stimuli and would gate new afferent signals from cortical and subcortical regions to update goal representations and behavioral strategies (Montague et al., 2004; Seamans and Yang, 2004; Daw et al., 2006; Durstewitz and Seamans, 2008; Ellwood et al., 2017).

### Development of the dopamine mesocortical pathway in early life and adolescence

In rodents, the first mesencephalic dopamine neurons appear during embryonic life (E12) and immediately start to extend projections to the developing telencephalic regions (Riddle and Pollock, 2003; Van den Heuvel and Pasterkamp, 2008; Islam et al., 2021). Dopamine fibers are detected in the striatum and the nucleus accumbens at E15-E17 (Voorn et al., 1988). The nigrostriatal and mesolimbic dopamine pathways end their development during the first postnatal week (Kalsbeek et al., 1988; Voorn et al., 1988). Dopamine fibers appear in the mPFC shortly after from E19 and continue to develop during postnatal life (Van Eden et al., 1987; Kalsbeek et al., 1988). In contrast to other neuromodulatory systems in the mPFC, such as noradrenaline or serotonin (Levitt and Moore, 1979; Lidov et al., 1980; Lambe et al., 2000; Naneix et al., 2012), the mesocortical dopamine pathway has a uniquely protracted maturation (Figure 1B). The density of dopamine fibers continues to increase until adulthood in rodents and primates in all mPFC subregions (Kalsbeek et al., 1988; Rosenberg and Lewis, 1994; Benes et al., 1996; Naneix et al., 2012; Hoops and Flores, 2017; Willing et al., 2017; Reynolds and Flores, 2021). This late development parallels increased basal dopamine levels in the mPFC during adolescence and decreased levels of dopamine metabolites (Nomura et al., 1976; Naneix et al., 2012) suggesting a broad increase in dopamine availability and action in prefrontal regions from childhood to adulthood. This is associated with an increase in the proportion of prefrontal cells, especially GABA neurons, in contact with dopamine fibers but also in the absolute number of dopamine varicosities per neuron (Benes et al., 1996), suggesting an increased dopaminergic modulation of these cells.

The continuous growth of dopamine fibers in prefrontal regions likely involves a combination of continued long-range axon growth from the VTA and increased sprouting from dopamine axons already present in the mPFC. There is now considerable evidence that these processes are under the control of several extracellular guidance cues including ephrins, semaphorins, netrins or slits during embryonic and postnatal stages (Van den Heuvel and Pasterkamp, 2008; Brignani and Pasterkamp, 2017; Hoops and Flores, 2017; Islam et al., 2021). The development of the mesocortical dopamine pathway during adolescence seems to be especially dependent of the Netrin-1/DCC (Hoops and Flores, 2017). Dopamine neurons express high levels of the Netrin-1 receptor during embryonic and early post-natal life (pre-weaning) which decrease toward adulthood (Manitt et al., 2010). A proportion of dopamine axons innervating the mPFC at adulthood are still growing through the nucleus accumbens during adolescence (Reynolds et al., 2015, 2018; Reynolds and Flores, 2021). This process is under the control of the Netrin-1 receptor and DCC. Reduced DCC expression in dopamine neurons increases dopamine innervation and dopamine levels in the mPFC (Manitt et al., 2011, 2013; Reynolds et al., 2018). These results suggest that high DCC-expressing dopamine neurons are attracted by brain regions expressing low levels of Netrin-1 such as the nucleus accumbens thus forming the mesolimbic pathway early in development (Manitt et al., 2011). In contrast, prefrontal neurons express high levels of Netrin-1 which attract a specific population of dopamine neurons expressing low levels of DCC during adolescence (Flores et al., 2005; Grant et al., 2007, 2009; Manitt et al., 2011; Reynolds et al., 2018). In addition to guidance cues the refinement of the dopamine system during adolescence may be under the influence of glial cells which contribute to synaptic development and pruning (for review see Béchade et al., 2013; Kettenmann et al., 2013; Brenhouse and Schwarz, 2016). Recent findings show that the downregulation of dopamine receptors in the nucleus accumbens during early adolescence is dependent of microglial activation, especially in males (Kopec et al., 2018). A similar mechanism within the mPFC remains to be demonstrated but microglia cells are already known to regulate the pruning of mPFC glutamatergic synapses during adolescence (Mallya et al., 2019; Blagburn-Blanco et al., 2022).

In addition to the growth of dopamine fibers the activity of dopamine cells also undergoes important changes during adolescence. *In vivo* and *ex vivo* recordings have revealed that VTA dopamine neurons in rats have higher tonic firing rates during adolescence compared to adulthood, peaking during late adolescence (PND40-50). In addition, although adolescent rats have a similar proportion of phasic activity episodes, these are consistently longer compared to those of adults (McCutcheon and Marinelli, 2009; McCutcheon et al., 2012; Marinelli and McCutcheon, 2014; but see Kim et al., 2016; McCane et al., 2021). It is currently unknown whether these age-related changes are specific to different defined neuronal populations or pathways. However, *in vivo* study in mice has demonstrated that the increase in dopamine neuron activity during adolescence may participate specifically to the development of the mesocortical pathway as the phasic activation of dopamine neurons in adolescents, but not in adults, promotes the formation of new synaptic boutons on mesocortical neurons (Mastwal et al., 2014). This is consistent with the role of dopamine in synaptogenesis also demonstrated *in vitro* (Parish et al., 2001; Fasano et al., 2008, 2010).

At the postsynaptic level, both PFC pyramidal glutamate neurons and GABA interneurons express dopamine D1-like (D1, D5) and D2-like (D2, D3, D4) receptors (Berger et al., 1991; Gaspar et al., 1995; Le Moine and Gaspar, 1998). Dopamine receptor expression during adolescence seems to present a complex reorganization in the cortex. However, data exploring this has produced somewhat conflicting and inconclusive results. For example, autoradiography studies in rats have shown either a steady increase in the expression of D1- and D2-like receptors (Tarazi and Baldessarini, 2000) or a binding peak at weaning followed by a decrease afterwards (Leslie et al., 1991). However immunohistochemistry (Andersen et al., 2000; Brenhouse et al., 2008; Brenhouse and Andersen, 2011) or qPCR studies (Naneix et al., 2012) have shown that the expression of dopamine receptors peaks during mid-adolescence, suggesting a short window of overexpression followed by pruning of dopamine synapses during late adolescence/early adulthood which may be related to the increased activity of dopamine neurons during the same period (McCutcheon and Marinelli, 2009; McCutcheon et al., 2012; Figure 1B). Recent advances in single cell RNA sequencing may clarify this picture by potentially showing different patterns of maturation within mPFC neuronal populations (Bhattacherjee et al., 2019; Tiklová et al., 2019).

## The endocannabinoid system and prefrontal circuits during adolescence

### The endocannabinoid system

Together with the dopamine system, the endocannabinoid (eCB) system is another central neuromodulator of executive functions (Egerton et al., 2006; Pattij et al., 2008) in adults and shares a similar late maturation during adolescence with the dopamine system (Caballero et al., 2016). In the following sections we will discuss how endocannabinoids and their endogenous receptors change across adolescence and how this influences the maturation of the PFC.

The two main endocannabinoids are 2-arachidonoylglycerol (2-AG) and N-arachidonoylethanolamine (AEA or anandamide; Mechoulam et al., 1995; Sugiura et al., 1995). They both bind to two key G-protein coupled receptors (GPCRs): cannabinoid receptor 1 (CB1)—expressed throughout the central nervous system (Herkenham et al., 1990; Mechoulam et al., 1995)—and cannabinoid receptor 2 (CB2) expressed predominantly in immune cells but also found in some neurons and glia (Atwood and Mackie, 2010; Zhang et al., 2014). We will focus here on the CB1 receptor due to its ubiquitous expression throughout the brain including the prefrontal cortex (Herkenham et al., 1990; Marsicano and Lutz, 1999) and its role in PFC development.

CB1 receptors are coupled to inhibitory G-proteins (G_i/o_ proteins) and their activation reduces cAMP levels, decreasing cAMP-dependent protein kinase activity (Howlett and Mukhopadhyay, 2000; Howlett, 2002). This induces the activation of A-type potassium channels, the inhibition of voltage-gated calcium channels and the disruption of neurotransmitter vesicles (Lovinger, 2008), which decreases the probability of neurotransmitter release. Unlike classical modulatory neurotransmitters which are stored in vesicles, endocannabinoids are synthesized and released *de novo* in times of sustained neuronal activity (Howlett and Mukhopadhyay, 2000; Freund et al., 2003) acting on presynaptic terminals (Wilson and Nicoll, 2001; Kreitzer and Regehr, 2002). This retrograde action allows neurons to filter and select afferent inputs *via* negative feedback. The principle psychoactive ingredient in cannabis, Δ9- tetrahydrocannabinol (THC), is a potent CB1 agonist (Gaoni and Mechoulam, 1964; Huestis et al., 2001).

Both 2-AG and AEA are derived from poly-unsaturated fatty acids but have distinct synthesis and metabolic pathways (Ueda et al., 2010; Ueda and Tsuboi, 2012). 2-AG is a major product of the phospholipase Cβ - diacylglycerol lipase pathway synthesized post-synaptically following sustained depolarization and by the subsequent increased calcium influx following activation of voltage-gated Ca^2+^ channels (Kano et al., 2009). 2-AG is metabolized by monoacylglycerol lipase (MAGL) localized mainly with pre-synaptic CB1 receptors (Häring et al., 2012). AEA synthesis on the other hand is a relatively minor biproduct of fatty acylethanolamine (N-acylethanolamines) species production triggered by increased intracellular calcium or cAMP levels in postsynaptic neurons (Bara et al., 2021). AEA is metabolized by fatty acid amide hydrolase (FAAH) localized post-synaptically to provide feedback inhibition (Häring et al., 2012).

Beyond their different synthetic and metabolic pathways, 2-AG and AEA also differ in terms of their pharmacological and functional actions *via* CB1 receptors. Pharmacologically 2-AG is a full agonist of the CB1 receptor, whereas AEA is a partial agonist (Sugiura et al., 1995, 2006). Despite binding to CB1 receptors with high affinity, AEA induces relatively poor intracellular signal transduction (Hillard et al., 1995). The slower time course of AEA's action makes it a good candidate for a tonic signal (Hill and Tasker, 2012) and thus AEA is implicated in long-term plasticity (Mackie, 2006). 2-AG on the other hand has a relatively low binding affinity for CB1 receptors but produces a much more robust intracellular response (Hillard et al., 1995). These dynamics combined with 2-AG release following sustained depolarization show a more characteristically phasic signal seen in shorter, activity-dependent synaptic plasticity (Lu and Mackie, 2016). In addition, 2-AG levels are generally several orders of magnitude higher than AEA levels in rodent brain tissue (Bisogno et al., 1999; Ueda and Tsuboi, 2012) which may be related to their different synthesis pathways.

Whilst we focus this review on the function and dysfunction of the eCB system in shaping the PFC through actions at CB1 receptors, it is important to consider that endocannabinoids also interact with other receptors and systems. Whilst predominantly expressed in peripheral tissues CB2 receptors are expressed in some microglia within the brain and their activation may have important effects we do not fully understand (Cabral et al., 2008). In addition, eCBs can activate or facilitate the activation of other receptors. For example, AEA is a TRPV1 receptor agonist and 2-AG can potentiate GABA-A receptors independently of CB1 receptor effects (Piscitelli and Di Marzo, 2012). In fact, eCB signaling has been shown to engage as many as 12 different receptors and involve many biosynthetic and degradative enzymes, transporters and other proteins diversifying the ways eCB signaling may impact local brain circuits (for a comprehensive discussion of the complex interactions between endocannabinoids and other receptors, transporters, and enzymes see Maccarrone, 2020).

### The eCB system in the developing brain

The CB1 receptor is the most abundant GPCR in the brain and the eCB system is uniquely positioned to have large scale influence on developing brain circuits. Our current understanding of the functioning and role the eCB system is still relatively new but we know this system is fundamentally important in all phases of brain development especially in controlling long-range axonal guidance and patterning of synaptic contacts (Berghuis et al., 2007; Harkany et al., 2007, 2008; Maccarrone et al., 2014; Bara et al., 2021). In cortical regions CB1 receptors are expressed at higher levels in GABAergic interneurons than in glutamatergic neurons with a higher expression in the PFC compared to motor and sensory cortices (Marsicano and Lutz, 1999). PFC CB1 expression in rodents peaks to its maximal density in early adolescence (Meyer et al., 2018), and then declines into adulthood (Figure 1B; Berrendero et al., 1999; Ellgren et al., 2008; Heng et al., 2011a; Peters et al., 2021c).

During adolescence 2AG and AEA levels also fluctuate. Within the PFC, AEA steadily increases from early to late adolescence, with concentrations reaching three times higher levels in late versus early adolescence before reaching stable levels into adulthood (Ellgren et al., 2008; Tirado-Muñoz et al., 2020). PFC 2-AG levels markedly decrease from early to late adolescence before stabilizing in adulthood (Ellgren et al., 2008; Rubino et al., 2015; Bara et al., 2021). These changes are accompanied by complementary changes in the activity of FAAH and MAGL (Long et al., 2012). Increased FAAH levels in adolescence may reflect in particular the tighter regulation of AEA during this key developmental period (Long et al., 2012).

Changes in the anatomical organization of the endocannabinoid system during adolescence are associated with important functional changes in synaptic plasticity which may also play an important role in long-range axonal pathfinding and patterning of synaptic contacts, both essential processes for prefrontal development (Berghuis et al., 2007; Maccarrone et al., 2014).

## Sex differences in the developing dopamine and endocannabinoid prefrontal systems

There are important sex differences in cortical development in humans, most prominent is the faster maturation of the cortex in females compared to males (Mutlu et al., 2013) as well as early differences in functional connectivity and sex specific age-related changes in resting state activity (Zuo et al., 2010). Similar sex differences are also reported in subcortical regions. Striatal development also occurs faster in females with striatal volume stabilizing around early adolescence, whereas in males this continues to develop until early adulthood (Raznahan et al., 2014; Hammerslag and Gulley, 2016). In rodents, key sex differences have been also reported in the development of the prefrontal cortex (Drzewiecki and Juraska, 2020). Sex and the onset of puberty, with changes in gonadal hormones during adolescence, may be critical factors in the late maturation of dopamine and endocannabinoid systems (Becker and Chartoff, 2019; Zachry et al., 2021). However, until recently most of the data from animal models has come from studies in males.

In the dopamine system there are crucial anatomical and functional sex differences in adulthood including a higher proportion of dopamine neurons in the VTA of female rodents and variation of striatal dopamine concentration with the estrous cycle through the expression of estrogen receptors in both dopamine and striatal GABAergic striatal neurons (for a recent complete review see Zachry et al., 2021). Within the mesocortical pathway in particular, female rats exhibit a higher proportion of dopamine neurons (tyrosine hydroxylase expressing cells) compared to males (>50 vs. 30%; Kritzer and Creutz, 2008). Estrogen receptors are also expressed at both presynaptic and postsynaptic level in the prefrontal cortex (Almey et al., 2015) and in different proportion between males and females (Kritzer and Creutz, 2008) but sex- and estrous cycle-related modulation of dopamine signaling in this region remains poorly understood. So far, studies have found no substantial sex differences in the time course of the development of mesocortical DA innervation (Willing et al., 2017). However, gonadectomy of male rats at adulthood or during the perinatal period alters the organization of the mesocortical dopamine pathway (Kritzer, 1998; Kritzer et al., 1999), suggesting there is a role of puberty in this maturation. Furthermore, there are clear age- and sex-dependent differences in subcortical dopamine release and reuptake in rats in adolescence and adulthood (Pitts et al., 2020). These differences remain to be investigated in the prefrontal cortex.

Consistent with the general earlier maturation of the female brain the increases in CB1 receptor density seen in adolescence begin earlier in female rodents compared to males (de Fonseca et al., 1993). The expression of CB1 receptors also varies across the estrous cycle in female mice and rats, with CB1 mRNA transcripts highest in diestrus and lowest on estrus (González et al., 2000). Levels of AEA are generally higher in female rats and again these fluctuate with the estrous cycle. In contrast, 2-AG does not seem to differ between males and females or as a function of the estrous cycle. In addition, eCB signaling can modulate gonadal hormones in both humans and rodents (Meyer et al., 2018). The relationship between puberty, sex hormones, neuromodulators and the maturation of brain functions during adolescence remains poorly understood and certainly requires a more in-depth investigation (Shansky and Murphy, 2021).

## Dopamine and endocannabinoid modulation of prefrontal circuits in the emergence of prefrontal functions during adolescence

Prefrontal circuits play a central role in action control including working memory processes (Goldman-Rakic et al., 2000; Seamans and Yang, 2004), goal-directed behaviors (Balleine and O'Doherty, 2010; Coutureau and Parkes, 2018; Turner and Parkes, 2020), response inhibition (Winstanley et al., 2006; Turner and Parkes, 2020), and behavioral flexibility (Floresco and Magyar, 2006). In adults, prefrontal dopamine (Floresco and Magyar, 2006; Winstanley et al., 2006) and endocannabinoid (Egerton et al., 2006; Pattij et al., 2008; McLaughlin et al., 2014; Gremel et al., 2016) signaling are important modulators of these executive functions. Several studies have reported alterations of these processes in adolescent rodents including adaptation to action-outcome contingency changes (Naneix et al., 2012, 2013), behavioral inhibition (Andrzejewski et al., 2011), attentional control (Burton and Fletcher, 2012), impulsivity (Doremus-Fitzwater et al., 2012), or behavioral extinction (Sturman et al., 2010). Conversely, adolescent rodents exhibit an increased sensitivity to rewards (Wilmouth and Spear, 2009; Friemel et al., 2010; Doremus-Fitzwater et al., 2012), environmental stimuli driving reward-seeking responses and motivated behaviors (Burton et al., 2011; Marshall et al., 2020), and impulsivity (Adriani and Laviola, 2003). These behavioral changes appear to be essential to the transition from childhood to adulthood by increasing exploration and novelty seeking but may also drive risk-taking behaviors.

**Figure 2:**
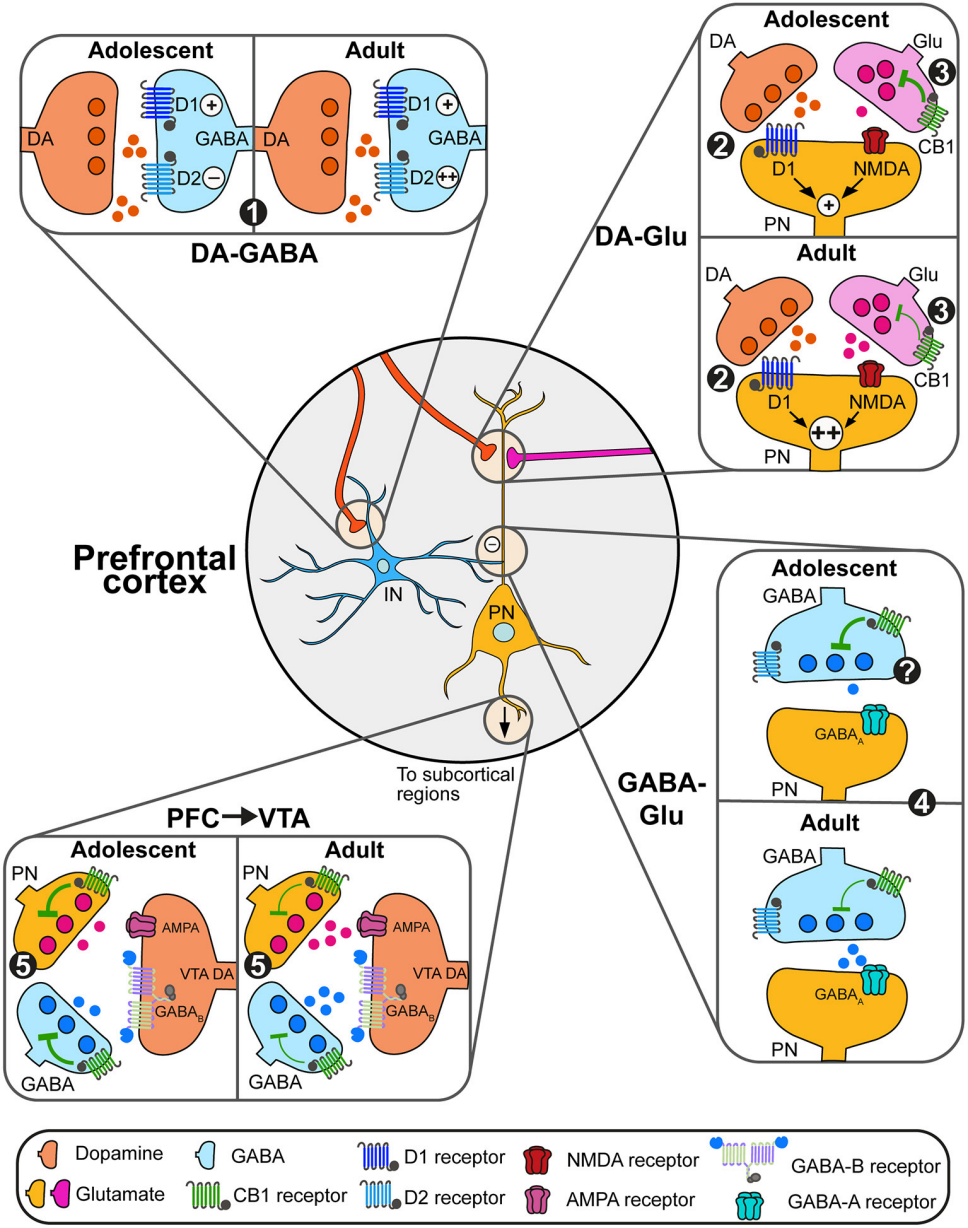
Adolescent changes in the modulation of prefrontal circuits by dopamine and endocannabinoids. (1) During adolescence, dopamine (DA, orange) increases the excitability of prefrontal GABAergic interneurons (blue) solely through D1 actions. At adulthood, dopamine facilitates interneurons excitability through both D1 and D2 receptors, which may change GABAergic control of pyramidal neuron (PN) activity. (2) Dopamine actions through D1 receptors enhance glutamatergic NMDA signaling on glutamate (Glu, pink and orange) neurons. This effect is more robust during late adolescence/adulthood, allowing the emergence of persistent depolarizations of pyramidal neurons (PN, orange). (3) eCB retrograde control (green) of glutamate transmission decreases between adolescence and adulthood, likely due to changes in expression levels of CB1 receptors. (4) At adulthood, the combined action of dopamine on D2 receptors and eCB signaling control the activity of plasticity of GABAergic synapses on PN. Changes in this process during adolescence remain to be demonstrated. (5) PN controls the activity of subcortical circuits. Within the VTA for instance, both PN glutamate transmission and local GABAergic transmission control the activity of DA neurons and DA release. Developmental changes in eCB signaling through the expression of CB1 receptors on PN and GABAergic neurons may change this regulation.

Prefrontal functions and physiology are the results of a complex balance between excitatory (glutamatergic) and inhibitory (GABAergic) transmission regulated by different neuromodulation system including the dopamine and endocannabinoid systems (O'Donnell, 2011; Caballero et al., 2016; Figure 2). In the following sections, we will focus on how developmental changes in prefrontal dopamine and eCB systems represent an important mechanism in the emergence of PFC functions by increasing both the stability of PFC microcircuits and the timed filtering of subcortical inputs essential to either maintain or switch behavioral strategies adapted to changing environments (Simon and Moghaddam, 2014; Reichelt, 2016).

### Dopamine and endocannabinoid modulation of prefrontal glutamatergic neurons

Both dopamine and endocannabinoids modulate the activity of pyramidal glutamate neurons and prefrontal glutamatergic plasticity. In adults, dopamine's action on prefrontal circuits is highly dependent of the excitability state of pyramidal neurons and interneurons. Low dopamine levels maintain prefrontal networks, mainly through actions on GABAergic interneurons and D2 receptors. Increases in dopamine levels during phasic activity recruits D1 receptors (Montague et al., 2004; Seamans and Yang, 2004; Durstewitz and Seamans, 2008) required for prefrontal functions such as executive functions (Floresco and Magyar, 2006; Floresco and Jentsch, 2011) and appetitive learning (Baldwin et al., 2002). Dopamine D1 signaling interacts with glutamatergic N-methyl-D-aspartate (NMDA) receptor signaling through D1-dependent mechanisms to induce long-lasting plateau depolarizations (up-state) of pyramidal cells (Tseng and O'Donnell, 2005). Similarly, CB1-dependent eCB signaling regulates prefrontal glutamatergic activity through depolarization-induced suppression of excitation process (Auclair et al., 2000; Kreitzer and Regehr, 2002; Fortin and Levine, 2007; Lovinger, 2008) and long-term depression in deep cortical layers (V–VI; Lafourcade et al., 2007).

As prefrontal D1 expression levels change during adolescence (Leslie et al., 1991; Tarazi and Baldessarini, 2000; Brenhouse et al., 2008; Naneix et al., 2012), *ex vivo* studies also showed that D1-NMDA interactions emerge during adolescence (Tseng and O'Donnell, 2005; Heng et al., 2011b; Flores-Barrera et al., 2014; Caballero et al., 2016). Despite important anatomical changes on both glutamatergic projections from the amygdala and the hippocampus to the PFC (Cunningham et al., 2002; Johnson et al., 2016; Calabro et al., 2020), only the latter presents a delayed functional maturation during adolescence (Caballero et al., 2014b; Flores-Barrera et al., 2014). Long-term potentiation (LTP) of hippocampal-prefrontal synapses on pyramidal only appear after PND50 in relationship with changes in the expression of GluN2B subunit composition of NMDA receptors. This process parallels the decrease of CB1-related inhibition of glutamatergic transmission (Heng et al., 2011a; Caballero et al., 2016) which may facilitate excitatory transmission from the PFC to subcortical circuits. Interestingly, anatomical studies have shown important changes in D1 expression levels in prefronto-accumbens neurons which may underlie increased incentive salience in response to environmental cues seen in adolescents (Brenhouse et al., 2008; Sonntag et al., 2014).

### Dopamine and endocannabinoid modulation of prefrontal GABAergic neurons

As for glutamatergic signaling, prefrontal GABAergic transmission is also still undergoing important developmental changes during adolescence. Previous studies demonstrated a progressive increase in the expression levels of calcium binding proteins parvalbumin and a parallel decrease in the expression of calretinin (Caballero et al., 2014a), increasing the proportion of fast-spiking interneurons. This process is dependent on NMDA glutamatergic transmission onto GABAergic interneurons (Flores-Barrera et al., 2014; Caballero et al., 2016; Bogart and O'Donnell, 2018) which may be related to the continuous development of projections from the hippocampus and the amygdala during this period (Cunningham et al., 2002; Johnson et al., 2016; Calabro et al., 2020).

Before adolescence, dopamine facilitates GABAergic transmission by increasing GABA interneurons excitability only through D1 receptor signaling (Gorelova et al., 2003; Tseng et al., 2006). Intriguingly, during late adolescence (PND50), dopamine increases GABA neurons excitability through both D1 and D2 signaling (Tseng et al., 2006; Tseng and O'Donnell, 2007; Caballero et al., 2016). This process is still poorly understood but may involve changes in the expression of D2 receptors and their signaling through β-arrestins which are able to enhance firing of parvalbumin interneurons in the cortex (Urs et al., 2016).

Less is known about the developmental changes of eCB-dependent regulation of GABAergic transmission. In adults, CB1 signaling suppresses GABA release onto glutamatergic cells (Chiu et al., 2010; Hill et al., 2011) and drives depolarization-induced suppression of inhibition at these synapses (Kiritoshi et al., 2013). Moreover, CB1 receptors also colocalize with dopamine D2 receptors on GABA cells, participating in the establishment of long-term depression of GABA synapses (Chiu et al., 2010). It is currently unknown if the changes in prefrontal CB1 receptor expression during adolescence follow the same pattern in glutamatergic and GABAergic neurons (Berrendero et al., 1999; Ellgren et al., 2008; Meyer et al., 2018; Peters et al., 2021c). As for glutamatergic pyramidal neurons, potential changes in CB1 and dopamine modulation of prefrontal GABAergic transmission may contribute to the establishment of excitation/inhibition balance, increasing the selection of specific inputs, decreasing the signal-to-noise ratio and refining prefrontal processing (Montague et al., 2004; Seamans and Yang, 2004; Durstewitz and Seamans, 2008; O'Donnell, 2011; Caballero et al., 2016).

### Beyond prefrontal microcircuits: Dopamine and endocannabinoid modulation of prefrontal signaling to subcortical regions

Changes in the prefrontal endocannabinoid system during adolescence may also impact the dopamine system and dopamine signaling in prefrontal subcortical targets, modulating dopamine-related behaviors such as reward prediction error, learning, motivation and event salience (Floresco and Magyar, 2006; Berridge, 2007; Schultz, 2007; Wickens et al., 2007; Bromberg-Martin et al., 2010; Berke, 2018).

In the VTA, dopamine neurons synthesize and release eCBs but do not express CB1 receptors (Julian et al., 2003), VTA eCBs act on local GABA interneurons as well as on GABA and glutamate terminals (Szabo et al., 2002; Melis et al., 2004; Riegel and Lupica, 2004; Wang et al., 2015). This results in the indirect disinhibition of dopamine neurons and the increase of dopamine release in terminal regions including the nucleus accumbens (Cheer et al., 2000, 2007; Oleson et al., 2012; Zlebnik and Cheer, 2016; Covey et al., 2017; Peters et al., 2021a,b) and the prefrontal cortex (Pistis et al., 2002). As in cortical regions, CB1 receptor expression in the VTA increases during early adolescence (PND 40) before decreasing to reach adult levels (de Fonseca et al., 1993) but the neuronal specificity of such changes remains unknown. This may be related to changes in CB1 levels on prefrontal glutamatergic terminals in the VTA controlling the activity of dopamine neurons (Carr and Sesack, 2000; Heidbreder and Groenewegen, 2003; Gabbott et al., 2005; Beier et al., 2015). These complex changes may represent an important mechanism underlying developmental changes in VTA responses to rewarding and aversive environmental stimuli (Kim et al., 2016; McCane et al., 2021) and dopamine-related behaviors (Spear, 2000).

Prefrontal glutamatergic neurons also strongly project to striatal regions (Vertes, 2004; Gabbott et al., 2005) to modulate action control processes (Alexander et al., 1986; Haber, 2003; Voorn et al., 2004). In the nucleus accumbens, cortical glutamatergic terminals control the activity of GABAergic medium spiny neurons (Surmeier et al., 2011) but also modulate local dopamine release through indirect actions on cholinergic interneurons (Cachope et al., 2012; Mateo et al., 2017). In adults, this cortical control of mesolimbic dopamine signal is dependent on CB1 receptors expressed on glutamatergic terminals. Combined with changes in the expression of dopamine D1 receptors by prefronto-accumbens neurons (Brenhouse et al., 2008; Sonntag et al., 2014), changes in CB1 expression in the same neurons may strongly change dopamine release in subcortical regions between adolescents and adults, impacting the control of motivated behaviors. Accordingly, *ex vivo* and *in vivo* studies have reported age-related changes in dopamine release in different striatal regions (Matthews et al., 2013; Pitts et al., 2020) and in striatal neuronal responses during instrumental and Pavlovian tasks (Galiñanes et al., 2009; Sturman and Moghaddam, 2012) which may also underlie enhanced reward sensitivity and executive functions deficits during adolescence.

## Vulnerability of prefrontal circuits during adolescence

Unfortunately, the late maturation of prefrontal circuits and the associated behavioral changes also creates a window of vulnerability to external insults which may have long-lasting deleterious impact on brain functions. Accordingly, adolescence is the period of life associated with the onset of numerous neuropsychiatric disorders in humans, such as schizophrenia, substance abuse or eating disorders (Paus et al., 2008). As previously stated, adolescents and the adolescent brain are especially sensitive to valence-based stimuli such as drugs of abuse, palatable foods or stressful experience (Spear, 2000; Andersen, 2003; Crews et al., 2007; Reichelt, 2016; Reichelt and Rank, 2017). In this last section, we will focus on two specific examples to illustrate how specific exposure during adolescence may impact prefrontal dopamine and eCB systems and related functions: the effects of cannabis (or CB1 agonists) and the effects of palatable foods.

### Impact of adolescent cannabinoid exposure on prefrontal circuits and functions

Cannabis is one of the most commonly used drugs of abuse, especially amongst adolescents (Miech et al., 2020). THC, the principal psychoactive constituent of cannabis, is a potent CB1 agonist (Gaoni and Mechoulam, 1964; Huestis et al., 2001). We do not fully understand how adolescent use in particular impacts the developing brain, however there is emerging evidence that altering the eCB system through such external insults may have important consequences especially at the prefrontal level (Schneider, 2008; Bara et al., 2021). Accordingly, numerous studies in humans and animal models report associations between adolescent cannabis exposure and increased risk of psychiatric conditions which a emerge in adolescence including psychosis, mood disorders and substance use disorders (Laviolette and Grace, 2006; Rubino et al., 2012; Renard et al., 2016).

Chronic CB1 agonist treatment in adolescent animals causes alterations in multiple PFC dependent behaviors, including anxiety (O'Shea et al., 2004), short- and long-term spatial and working memory (Rubino et al., 2009; Renard et al., 2013), motivational and emotional processes (Schneider and Koch, 2003), social behaviors (Schneider et al., 2008), and drug-seeking responses (Ellgren et al., 2007; Higuera-Matas et al., 2008). These alterations are specific to adolescence and are not observed when agonists are administered in adulthood. Importantly, the functioning and vulnerability of the eCB system presents important sex-differences that we do not discuss here (see Ginder et al., 2022 for a complete review of this topic) but which may play a key role in normal and pathological development during adolescence and puberty.

These behavioral phenotypes are associated with profound anatomical and functional alterations of prefrontal circuits (Rubino et al., 2015). These include alterations in mPFC CB1 receptor density and impaired eCB-mediated long-term depression (LTD) of prefrontal excitatory transmission (Rubino et al., 2015; Cuccurazzu et al., 2018). These changes are associated with an increased expression of GluA1 and GluN2B subunits in AMPA and NMDA receptors respectively (Rubino et al., 2015), altering the normal development of PFC glutamatergic transmission (Lovelace et al., 2015; Rubino et al., 2015). Interestingly, these effects are reversed by enhancing AEA levels at adulthood (Cuccurazzu et al., 2018), highlighting the importance of eCB signaling in normative prefrontal development and function.

In addition to its action on prefrontal glutamatergic transmission, there is emerging evidence that prefrontal GABA interneurons may be especially vulnerable to adolescent THC (or synthetic agonist) exposure (Cass et al., 2014; Renard et al., 2018; Peters et al., 2021c). Adolescent, but not adult, exposure to the synthetic CB1 agonist WIN55,212-2 decreases prefrontal GABA signaling resulting in dysregulation of cortical oscillatory activity (Cass et al., 2014; Renard et al., 2017). Conversely, activating prefrontal GABA-A receptors restores behavioral and dopaminergic abnormalities observed following adolescent THC exposure (Renard et al., 2017). Reduced prefrontal GABAergic transmission is associated with hyperdopaminergic state and alterations of prefrontal cortex-related behaviors (Yee et al., 2005; Enomoto et al., 2011), mimicking key behavioral alterations observed for instance in schizophrenia (Carlsson et al., 1997; O'Carroll, 2000; Nakazawa et al., 2012).

Adolescent cannabis exposure also directly impacts the activity of dopamine neurons in the VTA with potential alterations of eCB-mediated inhibition of VTA GABA neurons and glutamate terminals, resulting in a decrease of eCB-dependent disinhibition of dopamine neuron firing and dopamine release in the nucleus accumbens (Scherma et al., 2016), suggesting a broader impact of cannabis during adolescence on dopamine functions. The specific impact of adolescent THC exposure on mesocortical dopamine neurons or on dopamine release and signaling within the prefrontal cortex still needs to be investigated. Similarly, other drugs of abuse seem to have a critical influence on corticolimbic circuits and brain functions if they are used during adolescence compared to adulthood (for a complete view on the impact of drugs on adolescent brains see these recent books edited by Bell and Rahman, 2021a,b).

### Impact of adolescent palatable food overconsumption on prefrontal circuits and functions

In parallel to cannabis use by teenagers, in recent decades there has been a dramatic rise of obesity amongst children and adolescents, due to the increased availability of energy-dense and highly palatable foods and drinks (Crews et al., 2007; Wang et al., 2008; Ogden et al., 2016). Moreover, animal studies have shown that adolescents are more sensitive to palatable foods such as high-fat and high-sugar foods (Wilmouth and Spear, 2009; Friemel et al., 2010; Marshall et al., 2017, 2020) which may increase food-seeking behaviors, lead to overconsumption, unbalanced dietary habits and obesity.

The dopamine system is well-known to respond directly to food rewards and modulate food-seeking behaviors by acting in prefrontal and subcortical regions (Hajnal and Norgren, 2001; Bassareo et al., 2002; Hajnal et al., 2004; Berridge, 2007; Avena et al., 2008; de Araujo et al., 2010; Kenny, 2011; McCutcheon, 2015; Alhadeff et al., 2019; Zimmerman and Knight, 2020; Grove et al., 2022). Similarly, eCBs are tightly linked to feeding as they are derived from poly-unsaturated fatty acids and are able to modulate homeostatic and non-homeostatic/hedonic feeding (Mahler et al., 2007; Maccarrone et al., 2010; Lau et al., 2017; Coccurello and Maccarrone, 2018). In humans and rodents, obesity in adults is associated with alteration of both dopamine and eCB circuits (Berthoud and Morrison, 2008; South and Huang, 2008; Volkow et al., 2011; Bello et al., 2012) but also with dysfunction of prefrontal functions (Reichelt, 2016; Lowe et al., 2019). In adolescents, the delayed development of both dopamine and eCB systems may simultaneously support this adolescent sensitivity to overnutrition, but also provides a vulnerability window for the long-term effects of unbalanced dietary habits.

The differential time course in the development of cortical and subcortical dopamine pathways may be responsible of increased reactivity to palatable foods during adolescence, leading to overconsumption and increased food-seeking responses (Wilmouth and Spear, 2009; Friemel et al., 2010; Marshall et al., 2017, 2020). As eCB signaling controls the activity of mesocorticolimbic circuits, increased levels of CB1 receptors during adolescence may also participate to this process by decreasing VTA GABAergic activity and decreasing cortical control on VTA and striatal neurons, all together leading to increased dopamine activity (Lau et al., 2017; Sallam and Borgland, 2021) and food-seeking behaviors (Mateo et al., 2017).

Several studies have also investigated the impact of adolescent exposure to palatable food on related brain functions to understand how vulnerable these circuits may be to natural rewards. Adolescent overnutrition causes complex behavioral alterations which seem to be highly dependent on diet parameters including but not limited the constitution of the diet (e.g. high fat, high sugar, cafeteria diet), the duration of exposure (age at start, length of time exposed, withdrawal period between diet and testing) or other exposure parameters (e.g., continuous or limited access, combined access with control diet or not). We focus here on reward- and prefrontal-related functions but there is also an important literature on the effect of these dietary habits on learning, memory and emotional behaviors (for recent reviews on the topic see Kendig, 2014; Tsan et al., 2021). Taken together, several studies indicate that exposure to highly palatable foods during adolescence, but not at adulthood, strongly impacts reward-related functions including the preference for palatable foods (Vendruscolo et al., 2010b; Carlin et al., 2016; Naneix et al., 2016; Rabasa et al., 2016; Gueye et al., 2018), conditioned place preference for food (Privitera et al., 2011), motivation for food rewards (Frazier et al., 2008; Vendruscolo et al., 2010a; Reichelt et al., 2016; Tantot et al., 2017; Wong et al., 2017; Naneix et al., 2018; Ducrocq et al., 2019), hedonic processing (Naneix et al., 2016; but see Steele et al., 2019), and sensitivity to drugs of abuse (Blanco-Gandía et al., 2017; Naneix et al., 2017). More importantly, several adolescent dietary habits also seem to impact prefrontal-related functions including control of goal-directed behavior (Tantot et al., 2017; but see Kendig et al., 2013), memory extinction (Baker and Reichelt, 2016), and cognitive control (Reichelt et al., 2015; Labouesse et al., 2017; Robertson and Rasmussen, 2017; Steele et al., 2019).

At the neurobiological level, exposure to highly palatable foods during adolescence is associated with broad anatomical and functional alterations of the dopamine system including reduced dopamine clearance in the striatum (Baladi et al., 2015), increased sensitivity of the mesolimbic pathway (Naneix et al., 2017) and changes in the expression of dopamine markers (Teegarden et al., 2009; Carlin et al., 2016; Naneix et al., 2017, 2018) in the prefrontal cortex and the nucleus accumbens. To our knowledge, the specific effects of such adolescent diets on prefrontal dopamine release, transmission and associated plasticity has not been investigated. However, the functioning of prefrontal microcuircuits is strongly impacted by adolescent consumption of palatable foods. As with cannabis exposure, prefrontal GABA signaling seems especially vulnerable, with diet-induced decreased of GABA levels (Sandoval-Salazar et al., 2016) and numbers of parvalbumin interneurons (Reichelt et al., 2015, 2019; Baker and Reichelt, 2016). Moreover, juvenile obesity in mice also impairs prefrontal glutamatergic transmission including NMDA-dependent synaptic plasticity and AMPA postsynaptic currents (Labouesse et al., 2017). Surprisingly, given its role in feeding and appetite, there are no clear data available on the impact of such diet exposure on the eCB system. In adult animals, obesity or the exposure to high-sugar/high-fat foods alters eCB-dependent cortical synaptic plasticity and insulin effects on VTA dopamine neurons to control ingestive behaviors (Labouèbe et al., 2013; Liu and Borgland, 2015; Liu et al., 2016; Lau et al., 2017). As maternal high-fat diet dysregulates the eCB system (Almeida et al., 2022; Urbonaite et al., 2022), we might predict high-fat / high-sugar diet exposure during adolescence would have similar effects. The exact nature of these alterations is not well-understood and the mechanisms which make adolescent PFC circuits particularly vulnerable are yet to be determined. However, altered dopamine and eCB signaling may both contribute to long-lasting diet-induced dysfunctions in executive functions.

## Conclusion

Dopamine and eCB signaling are central elements in the regulation of prefrontal microcircuits and their dysregulation is associated with numerous neuropsychiatric disorders that typically first present in adolescence. During adolescence, prefrontal circuits undergo important developmental changes which support the emergence of adaptive adult behaviors. Dopamine and eCB prefrontal systems have a similar delayed maturation during adolescence characterized by transitory increases in receptors expression, gradual increases in neurotransmitter levels and important changes in synaptic effects. Given their interactions with different neuronal populations and effects at different synaptic sites, adolescent remodeling of these systems may play a key role in the successful maturation of prefrontal circuits and their functioning.

Despite a considerable increase in research on this topic during the last few decades, numerous questions remain. By increasing the recruitment of GABAergic interneurons and the sustained activity of pyramidal neurons during the transition to adulthood, prefrontal dopamine signaling increases the selection of specific inputs, decreasing the signal-to-noise ratio and refining prefrontal processing (O'Donnell, 2011; Caballero et al., 2016). Specific guidance cues control the late development of dopamine fibers (Hoops and Flores, 2017) but the mechanisms governing synaptic changes and the emergence of adult-like PFC activity compared to limbic regions remains unclear. Moreover, despite clear effects of external insults (drugs of abuse, palatable foods, and stress) during adolescence on prefrontal functions, anatomical organization of the mesocortical pathway and on the functioning of prefrontal glutamate and GABA transmission, the impact on the functioning of this dopamine pathway *in vivo* and its interaction with local microcircuits remains poorly explored compared to other dopamine circuits. One of the reasons for this may come from the unique properties of the mesocortical pathway (Sesack et al., 1998; Lammel et al., 2008) which have made it challenging to investigate *in vivo* until recently.

For the eCB system, most of our knowledge on the maturation of the eCB system comes from studying the effect of drugs including THC or CB1 agonists given during adolescence. The prefrontal eCB system presents important anatomical changes during adolescence (Rubino et al., 2015; Caballero et al., 2016). However, little is known about related neurophysiological changes on different prefrontal populations and the underlying processes governing these changes. Considering the important role of eCB in responses to food and in the control of feeding (Lau et al., 2017), there is also an important lack of information on the impact of unbalanced dietary habits during adolescence on eCB signaling.

Finally, a crucial point to consider is how dopamine and eCB systems interact through development. At adulthood they exhibit strong functional interactions to control diverse motivated behaviors. As they both exhibit a continued maturation throughout adolescence, which is not observed in other neuromodulatory systems, they may represent a crucial milestone in postnatal brain development in health and disease. The recent development of cutting-edge tools such as specific optical sensors for *in vivo* and *ex vivo* recordings (Labouesse et al., 2020; Dong et al., 2021) allows us to dissect microcircuits and use longitudinal studies to measure dynamic changes of prefrontal dopamine and eCB signaling from childhood to adulthood which until now were not possible.

## Author contributions

All authors conceptualized, wrote and edited the manuscript, designed and produced all figures, and approved the submitted version.

## Funding

FN was funded by the Royal Society (RGS_R1_211013), the Wellcome Trust Institutional Strategic Support Fund (RG13793-43), and the Tenovus Scotland (G21.11). KZP was funded by the Medical Research Council (project MR/T03260X/1) at the University of Sussex in the lab of Eisuke Koya.

## Conflict of interest

The authors declare that the research was conducted in the absence of any commercial or financial relationships that could be construed as a potential conflict of interest.

## Publisher's note

All claims expressed in this article are solely those of the authors and do not necessarily represent those of their affiliated organizations, or those of the publisher, the editors and the reviewers. Any product that may be evaluated in this article, or claim that may be made by its manufacturer, is not guaranteed or endorsed by the publisher.

## References

[B1] Abercrombie E. D. Keefe K. A. DiFrischia D. S. Zigmond M. J. (1989). Differential effect of stress on *in vivo* dopamine release in striatum, nucleus accumbens, and medial frontal cortex. J. Neurochem. 52, 1655–1658. 10.1111/j.1471-4159.1989.tb09224.x 2709017

[B2] Adriani W. Laviola G. (2003). Elevated levels of impulsivity and reduced place conditioning with d-amphetamine: two behavioral features of adolescence in mice. Behav. Neurosci. 117, 695–703. 10.1037/0735-7044.117.4.695 12931955

[B3] Ahn S. Phillips A. G. (1999). Dopaminergic correlates of sensory-specific satiety in the medial prefrontal cortex and nucleus accumbens of the rat. J. Neurosci. Off. J. Soc. Neurosci. 19, RC29. 10.1523/JNEUROSCI.19-19-j0003.1999 10493774 PMC6782999

[B4] Alexander G. E. DeLong M. R. Strick P. L. (1986). Parallel organization of functionally segregated circuits linking basal ganglia and cortex. Annu. Rev. Neurosci. 9, 357–381. 10.1146/annurev.ne.09.030186.002041 3085570

[B5] Alhadeff A. L. Goldstein N. Park O. Klima M. L. Vargas A. Betley J. N. (2019). Natural and drug rewards engage distinct pathways that converge on coordinated hypothalamic and reward circuits. Neuron 103, 891–908.e6. 10.1016/j.neuron.2019.05.050 31277924 PMC6728176

[B6] Almeida M. M. Dias-Rocha C. P. Calviño C. Trevenzoli I. H. (2022). Lipid endocannabinoids in energy metabolism, stress and developmental programming. Mol. Cell. Endocrinol. 542, 111522. 10.1016/j.mce.2021.111522 34843899

[B7] Almey A. Milner T. A. Brake W. G. (2015). Estrogen receptors in the central nervous system and their implication for dopamine-dependent cognition in females. Horm. Behav. 74, 125–138. 10.1016/j.yhbeh.2015.06.010 26122294 PMC4820286

[B8] Andersen S. L. (2003). Trajectories of brain development: point of vulnerability or window of opportunity? Neurosci. Biobehav. Rev. 27, 3–18. 10.1016/S0149-7634(03)00005-8 12732219

[B9] Andersen S. L. Thompson A. T. Rutstein M. Hostetter J. C. Teicher M. H. (2000). Dopamine receptor pruning in prefrontal cortex during the periadolescent period in rats. Synap. N. Y. N 37, 167–169. 10.1002/1098-2396(200008)37:2<167::AID-SYN11>3.0.CO;2-B 10881038

[B10] Andrzejewski M. E. Schochet T. L. Feit E. C. Harris R. McKee B. L. Kelley A. E. (2011). A comparison of adult and adolescent rat behavior in operant learning, extinction, and behavioral inhibition paradigms. Behav. Neurosci. 125, 93–105. 10.1037/a0022038 21319891

[B11] Atwood B. K. Mackie K. (2010). CB2: a cannabinoid receptor with an identity crisis. Br. J. Pharmacol. 160, 467–479. 10.1111/j.1476-5381.2010.00729.x 20590558 PMC2931549

[B12] Auclair N. Otani S. Soubrie P. Crepel F. (2000). Cannabinoids modulate synaptic strength and plasticity at glutamatergic synapses of rat prefrontal cortex pyramidal neurons. J. Neurophysiol. 83, 3287–3293. 10.1152/jn.2000.83.6.3287 10848548

[B13] Avena N. M. Rada P. Hoebel B. G. (2008). Evidence for sugar addiction: behavioral and neurochemical effects of intermittent, excessive sugar intake. Neurosci. Biobehav. Rev. 32, 20–39. 10.1016/j.neubiorev.2007.04.019 17617461 PMC2235907

[B14] Baker K. D. Reichelt A. C. (2016). Impaired fear extinction retention and increased anxiety-like behaviours induced by limited daily access to a high-fat/high-sugar diet in male rats: implications for diet-induced prefrontal cortex dysregulation. Neurobiol. Learn. Mem. 136, 127–138. 10.1016/j.nlm.2016.10.002 27720810

[B15] Baladi M. G. Horton R. E. Owens W. A. Daws L. C. France C. P. (2015). Eating high fat chow decreases dopamine clearance in adolescent and adult male rats but selectively enhances the locomotor stimulating effects of cocaine in adolescents. Int. J. Neuropsychopharmacol. 18, pyv024. 10.1093/ijnp/pyv024 25805560 PMC4540111

[B16] Baldwin A. E. Sadeghian K. Kelley A. E. (2002). Appetitive instrumental learning requires coincident activation of NMDA and dopamine D1 receptors within the medial prefrontal cortex. J. Neurosci. Off. J. Soc. Neurosci. 22, 1063–1071. 10.1523/JNEUROSCI.22-03-01063.2002 11826135 PMC6758518

[B17] Balleine B. W. O'Doherty J. P. (2010). Human and rodent homologies in action control: corticostriatal determinants of goal-directed and habitual action. Neuropsychopharmacol. Off. Publ. Am. Coll. Neuropsychopharmacol. 35, 48–69. 10.1038/npp.2009.131 19776734 PMC3055420

[B18] Bara A. Ferland J.-M. N. Rompala G. Szutorisz H. Hurd Y. L. (2021). Cannabis and synaptic reprogramming of the developing brain. Nat. Rev. Neurosci. 22, 423–438. 10.1038/s41583-021-00465-5 34021274 PMC8445589

[B19] Bassareo V. De Luca M. A. Di Chiara G. (2002). Differential expression of motivational stimulus properties by dopamine in nucleus accumbens shell vs. core and prefrontal cortex. J. Neurosci. Off. J. Soc. Neurosci. 22, 4709–4719. 10.1523/JNEUROSCI.22-11-04709.2002 PMC6758788 12040078

[B20] Béchade C. Cantaut-Belarif Y. Bessis A. (2013). Microglial control of neuronal activity. Front. Cell. Neurosci. 7, 32. 10.3389/fncel.2013.00032 23543873 PMC3610058

[B21] Becker J. B. Chartoff E. (2019). Sex differences in neural mechanisms mediating reward and addiction. Neuropsychopharmacol. Off. Publ. Am. Coll. Neuropsychopharmacol. 44, 166–183. 10.1038/s41386-018-0125-6 29946108 PMC6235836

[B22] Beier K. T. Steinberg E. E. DeLoach K. E. Xie S. Miyamichi K. Schwarz L.. (2015). Circuit architecture of VTA dopamine neurons revealed by systematic input-output mapping. Cell 162, 622–634. 10.1016/j.cell.2015.07.015 26232228 PMC4522312

[B23] Bell R. L. Rahman S. (2021a). Effects of Peri-Adolescent Licit and Illicit Drug Use on the Developing CNS Part I. 1st Edn. Cambridge, MA: Academic Press.

[B24] Bell R. L. Rahman S. (2021b). Effects of Peri-Adolescent Licit and Illicit Drug Use on the Developing CNS: Part II. 1st Edn. Cambridge, MA: Academic Press. 10.1080/21622965.2022.2100821

[B25] Bello N. T. Coughlin J. W. Redgrave G. W. Ladenheim E. E. Moran T. H. Guarda A. S. (2012). Dietary conditions and highly palatable food access alter rat cannabinoid receptor expression and binding density. Physiol. Behav. 105, 720–726. 10.1016/j.physbeh.2011.09.021 22005165 PMC3621143

[B26] Benes F. M. Vincent S. L. Molloy R. Khan Y. (1996). Increased interaction of dopamine-immunoreactive varicosities with GABA neurons of rat medial prefrontal cortex occurs during the postweanling period. Synap. N. Y. N 23, 237–245. 10.1002/(SICI)1098-2396(199608)23:4<237::AID-SYN1>3.0.CO;2-8 8855508

[B27] Berger B. Gaspar P. Verney C. (1991). Dopaminergic innervation of the cerebral cortex: unexpected differences between rodents and primates. Trends Neurosci. 14, 21–27. 10.1016/0166-2236(91)90179-X 1709528

[B28] Berghuis P. Rajnicek A. M. Morozov Y. M. Ross R. A. Mulder J. Urbán G. M.. (2007). Hardwiring the brain: endocannabinoids shape neuronal connectivity. Science 316, 1212–1216. 10.1126/science.1137406 17525344

[B29] Berke J. D. (2018). What does dopamine mean? Nat. Neurosci. 21, 787–793. 10.1038/s41593-018-0152-y 29760524 PMC6358212

[B30] Berrendero F. Sepe N. Ramos J. A. Di Marzo V. Fernández-Ruiz J. J. (1999). Analysis of cannabinoid receptor binding and mRNA expression and endogenous cannabinoid contents in the developing rat brain during late gestation and early postnatal period. Synap. N. Y. N 33, 181–191. 10.1002/(SICI)1098-2396(19990901)33:3<181::AID-SYN3>3.0.CO;2-R 10420166

[B31] Berridge K. C. (2007). The debate over dopamine's role in reward: the case for incentive salience. Psychopharmacology 191, 391–431. 10.1007/s00213-006-0578-x 17072591

[B32] Berthoud H.-R. Morrison C. (2008). The brain, appetite, and obesity. Annu. Rev. Psychol. 59, 55–92. 10.1146/annurev.psych.59.103006.093551 18154499

[B33] Bhattacherjee A. Djekidel M. N. Chen R. Chen W. Tuesta L. M. Zhang Y. (2019). Cell type-specific transcriptional programs in mouse prefrontal cortex during adolescence and addiction. Nat. Commun. 10, 4169. 10.1038/s41467-019-12054-3 31519873 PMC6744514

[B34] Bisogno T. Berrendero F. Ambrosino G. Cebeira M. Ramos J. A. Fernandez-Ruiz J. J.. (1999). Brain regional distribution of endocannabinoids: implications for their biosynthesis and biological function. Biochem. Biophys. Res. Commun. 256, 377–380. 10.1006/bbrc.1999.0254 10079192

[B35] Björklund A. Dunnett S. B. (2007). Dopamine neuron systems in the brain: an update. Trends Neurosci. 30, 194–202. 10.1016/j.tins.2007.03.006 17408759

[B36] Blagburn-Blanco S. V. Chappell M. S. De Biase L. M. DeNardo L. A. (2022). Synapse-specific roles for microglia in development: new horizons in the prefrontal cortex. Front. Mol. Neurosci. 15, 965756. 10.3389/fnmol.2022.965756 36003220 PMC9394540

[B37] Blakemore S.-J. (2012). Imaging brain development: the adolescent brain. NeuroImage 61, 397–406. 10.1016/j.neuroimage.2011.11.080 22178817

[B38] Blakemore S.-J. Robbins T. W. (2012). Decision-making in the adolescent brain. Nat. Neurosci. 15, 1184–1191. 10.1038/nn.3177 22929913

[B39] Blanco-Gandía M. C. Cantacorps L. Aracil-Fernández A. Montagud-Romero S. Aguilar M. A. Manzanares J.. (2017). Effects of bingeing on fat during adolescence on the reinforcing effects of cocaine in adult male mice. Neuropharmacology 113, 31–44. 10.1016/j.neuropharm.2016.09.020 27666001

[B40] Bogart L. J. O'Donnell P. (2018). Multiple long-range inputs evoke NMDA currents in prefrontal cortex fast-spiking interneurons. Neuropsychopharmacol. Off. Publ. Am. Coll. Neuropsychopharmacol. 43, 2101–2108. 10.1038/s41386-018-0029-5 29483660 PMC6098111

[B41] Brenhouse H. C. Andersen S. L. (2011). Developmental trajectories during adolescence in males and females: a cross-species understanding of underlying brain changes. Neurosci. Biobehav. Rev. 35, 1687–1703. 10.1016/j.neubiorev.2011.04.013 21600919 PMC3134153

[B42] Brenhouse H. C. Schwarz J. M. (2016). Immunoadolescence: neuroimmune development and adolescent behavior. Neurosci. Biobehav. Rev. 70, 288–299. 10.1016/j.neubiorev.2016.05.035 27260127 PMC5412135

[B43] Brenhouse H. C. Sonntag K. C. Andersen S. L. (2008). Transient D1 dopamine receptor expression on prefrontal cortex projection neurons: relationship to enhanced motivational salience of drug cues in adolescence. J. Neurosci. Off. J. Soc. Neurosci. 28, 2375–2382. 10.1523/JNEUROSCI.5064-07.2008 18322084 PMC4028226

[B44] Brignani S. Pasterkamp R. J. (2017). Neuronal subset-specific migration and axonal wiring mechanisms in the developing midbrain dopamine system. Front. Neuroanat. 11, 55. 10.3389/fnana.2017.00055 28740464 PMC5502286

[B45] Bromberg-Martin E. S. Matsumoto M. Hikosaka O. (2010). Dopamine in motivational control: rewarding, aversive, and alerting. Neuron 68, 815–834. 10.1016/j.neuron.2010.11.022 21144997 PMC3032992

[B46] Burton C. L. Fletcher P. J. (2012). Age and sex differences in impulsive action in rats: the role of dopamine and glutamate. Behav. Brain Res. 230, 21–33. 10.1016/j.bbr.2012.01.046 22326372

[B47] Burton C. L. Noble K. Fletcher P. J. (2011). Enhanced incentive motivation for sucrose-paired cues in adolescent rats: possible roles for dopamine and opioid systems. Neuropsychopharmacol. Off. Publ. Am. Coll. Neuropsychopharmacol. 36, 1631–1643. 10.1038/npp.2011.44 21508935 PMC3138669

[B48] Caballero A. Flores-Barrera E. Cass D. K. Tseng K. Y. (2014a). Differential regulation of parvalbumin and calretinin interneurons in the prefrontal cortex during adolescence. Brain Struct. Funct. 219, 395–406. 10.1007/s00429-013-0508-8 23400698 PMC3665762

[B49] Caballero A. Granberg R. Tseng K. Y. (2016). Mechanisms contributing to prefrontal cortex maturation during adolescence. Neurosci. Biobehav. Rev. 70, 4–12. 10.1016/j.neubiorev.2016.05.013 27235076 PMC5074870

[B50] Caballero A. Thomases D. R. Flores-Barrera E. Cass D. K. Tseng K. Y. (2014b). Emergence of GABAergic-dependent regulation of input-specific plasticity in the adult rat prefrontal cortex during adolescence. Psychopharmacology 231, 1789–1796. 10.1007/s00213-013-3216-4 23907651 PMC3873346

[B51] Cabral G. A. Raborn E. S. Griffin L. Dennis J. Marciano-Cabral F. (2008). CB2 receptors in the brain: role in central immune function. Br. J. Pharmacol. 153, 240–251. 10.1038/sj.bjp.0707584 18037916 PMC2219530

[B52] Cachope R. Mateo Y. Mathur B. N. Irving J. Wang H.-L. Morales M.. (2012). Selective activation of cholinergic interneurons enhances accumbal phasic dopamine release: setting the tone for reward processing. Cell Rep. 2, 33–41. 10.1016/j.celrep.2012.05.011 22840394 PMC3408582

[B53] Calabro F. J. Murty V. P. Jalbrzikowski M. Tervo-Clemmens B. Luna B. (2020). Development of hippocampal–prefrontal cortex interactions through adolescence. Cereb. Cortex 30, 1548–1558. 10.1093/cercor/bhz186 31670797 PMC7132933

[B54] Carlén M. (2017). What constitutes the prefrontal cortex? Science 358, 478–482. 10.1126/science.aan8868 29074767

[B55] Carlin J. L. McKee S. E. Hill-Smith T. Grissom N. M. George R. Lucki I.. (2016). Removal of high-fat diet after chronic exposure drives binge behavior and dopaminergic dysregulation in female mice. Neuroscience 326, 170–179. 10.1016/j.neuroscience.2016.04.002 27063418 PMC6922047

[B56] Carlsson A. Hansson L. O. Waters N. Carlsson M. L. (1997). Neurotransmitter aberrations in schizophrenia: new perspectives and therapeutic implications. Life Sci. 61, 75–94. 10.1016/S0024-3205(97)00228-2 9217267

[B57] Carr D. B. Sesack S. R. (2000). Projections from the rat prefrontal cortex to the ventral tegmental area: target specificity in the synaptic associations with mesoaccumbens and mesocortical neurons. J. Neurosci. Off. J. Soc. Neurosci. 20, 3864–3873. 10.1523/JNEUROSCI.20-10-03864.2000 PMC6772693 10804226

[B58] Casey B. J. Jones R. M. Hare T. A. (2008). The adolescent brain. Ann. N. Y. Acad. Sci. 1124, 111–126. 10.1196/annals.1440.010 18400927 PMC2475802

[B59] Cass D. K. Flores-Barrera E. Thomases D. R. Vital W. F. Caballero A. Tseng K. Y. (2014). CB1 cannabinoid receptor stimulation during adolescence impairs the maturation of GABA function in the adult rat prefrontal cortex. Mol. Psychiatry 19, 536–543. 10.1038/mp.2014.14 24589887 PMC3999247

[B60] Cass W. A. Gerhardt G. A. (1995). *In vivo* assessment of dopamine uptake in rat medial prefrontal cortex: comparison with dorsal striatum and nucleus accumbens. J. Neurochem. 65, 201–207. 10.1046/j.1471-4159.1995.65010201.x 7790861

[B61] Cheer J. F. Aragona B. J. Heien M. L. A. V. Seipel A. T. Carelli R. M. Wightman R. M. (2007). Coordinated accumbal dopamine release and neural activity drive goal-directed behavior. Neuron 54, 237–244. 10.1016/j.neuron.2007.03.021 17442245

[B62] Cheer J. F. Kendall D. A. Marsden C. A. (2000). Cannabinoid receptors and reward in the rat: a conditioned place preference study. Psychopharmacology 151, 25–30. 10.1007/s002130000481 10958113

[B63] Chiu C. Q. Puente N. Grandes P. Castillo P. E. (2010). Dopaminergic modulation of endocannabinoid-mediated plasticity at GABAergic synapses in the prefrontal cortex. J. Neurosci. Off. J. Soc. Neurosci. 30, 7236–7248. 10.1523/JNEUROSCI.0736-10.2010 20505090 PMC2905527

[B64] Coccurello R. Maccarrone M. (2018). Hedonic eating and the “delicious circle”: from lipid-derived mediators to brain dopamine and back. Front. Neurosci. 12, 271. 10.3389/fnins.2018.00271 29740277 PMC5928395

[B65] Coutureau E. Parkes S. L. (2018). Cortical determinants of goal-directed behavior, in Goal-Directed Decision Making, eds Morris R. Bornstein A. Shenhav A. (Cambridge, MA: Academic Press), 179–197. 10.1016/B978-0-12-812098-9.00008-5

[B66] Covey D. Mateo Y. Sulzer D. Cheer J. F. Lovinger D. M. (2017). Endocannabinoid modulation of dopamine neurotransmission. Neuropharmacology 124, 52–61. 10.1016/j.neuropharm.2017.04.033 28450060 PMC5608040

[B67] Crews F. He J. Hodge C. (2007). Adolescent cortical development: a critical period of vulnerability for addiction. Pharmacol. Biochem. Behav. 86, 189–199. 10.1016/j.pbb.2006.12.001 17222895 PMC11646682

[B68] Cuccurazzu B. Zamberletti E. Nazzaro C. Prini P. Trusel M. Grilli M.. (2018). Adult cellular neuroadaptations induced by adolescent THC exposure in female rats are rescued by enhancing anandamide signaling. Int. J. Neuropsychopharmacol. 21, 1014–1024. 10.1093/ijnp/pyy057 29982505 PMC6209859

[B69] Cunningham M. G. Bhattacharyya S. Benes F. M. (2002). Amygdalo-cortical sprouting continues into early adulthood: Implications for the development of normal and abnormal function during adolescence. J. Comp. Neurol. 453, 116–130. 10.1002/cne.10376 12373778

[B70] Daw N. D. O'Doherty J. P. Dayan P. Seymour B. Dolan R. J. (2006). Cortical substrates for exploratory decisions in humans. Nature 441, 876–879. 10.1038/nature04766 16778890 PMC2635947

[B71] de Araujo I. E. Ren X. Ferreira J. G. (2010). Metabolic sensing in brain dopamine systems. Results Probl. Cell Differ. 52, 69–86. 10.1007/978-3-642-14426-4_7 20865373

[B72] de Fonseca F. R. Ramos J. A. Bonnin A. Fernández-Ruiz J. J. (1993). Presence of cannabinoid binding sites in the brain from early postnatal ages. NeuroReport 4, 135–138. 10.1097/00001756-199302000-00005 8453049

[B73] Delevich K. Klinger M. Okada N. J. Wilbrecht L. (2021). Coming of age in the frontal cortex: the role of puberty in cortical maturation. Semin. Cell Dev. Biol. 118, 64–72. 10.1016/j.semcdb.2021.04.021 33985902 PMC12018213

[B74] Dong A. He K. Dudok B. Farrell J. S. Guan W. Liput D. J.. (2021). A fluorescent sensor for spatiotemporally resolved imaging of endocannabinoid dynamics *in vivo*. Nat. Biotechnol. 2021, 329169. 10.1101/2020.10.08.329169 34764491 PMC9091059

[B75] Doremus-Fitzwater T. L. Barreto M. Spear L. P. (2012). Age-related differences in impulsivity among adolescent and adult Sprague-Dawley rats. Behav. Neurosci. 126, 735–741. 10.1037/a0029697 22889309 PMC3583377

[B76] Drzewiecki C. M. Juraska J. M. (2020). The structural reorganization of the prefrontal cortex during adolescence as a framework for vulnerability to the environment. Pharmacol. Biochem. Behav. 199, 173044. 10.1016/j.pbb.2020.173044 33035531

[B77] Ducrocq F. Hyde A. Fanet H. Oummadi A. Walle R. De Smedt-Peyrusse V.. (2019). Decrease in operant responding under obesogenic diet exposure is not related to deficits in incentive or hedonic processes. Obes. Silver Spring Md 27, 255–263. 10.1002/oby.22358 30597761

[B78] Durstewitz D. Seamans J. K. (2008). The dual-state theory of prefrontal cortex dopamine function with relevance to catechol-o-methyltransferase genotypes and schizophrenia. Biol. Psychiatry 64, 739–749. 10.1016/j.biopsych.2008.05.015 18620336

[B79] Egerton A. Allison C. Brett R. R. Pratt J. A. (2006). Cannabinoids and prefrontal cortical function: insights from preclinical studies. Neurosci. Biobehav. Rev. 30, 680–695. 10.1016/j.neubiorev.2005.12.002 16574226

[B80] Ellgren M. Artmann A. Tkalych O. Gupta A. Hansen H. S. Hansen S. H.. (2008). Dynamic changes of the endogenous cannabinoid and opioid mesocorticolimbic systems during adolescence: THC effects. Eur. Neuropsychopharmacol. 18, 826–834. 10.1016/j.euroneuro.2008.06.009 18674887 PMC2745315

[B81] Ellgren M. Spano S. M. Hurd Y. L. (2007). Adolescent cannabis exposure alters opiate intake and opioid limbic neuronal populations in adult rats. Neuropsychopharmacol. Off. Publ. Am. Coll. Neuropsychopharmacol. 32, 607–615. 10.1038/sj.npp.1301127 16823391

[B82] Ellwood I. T. Patel T. Wadia V. Lee A. T. Liptak A. T. Bender K. J.. (2017). Tonic or phasic stimulation of dopaminergic projections to prefrontal cortex causes mice to maintain or deviate from previously learned behavioral strategies. J. Neurosci. Off. J. Soc. Neurosci. 37, 8315–8329. 10.1523/JNEUROSCI.1221-17.2017 28739583 PMC5577850

[B83] Enomoto T. Tse M. T. Floresco S. B. (2011). Reducing prefrontal gamma-aminobutyric acid activity induces cognitive, behavioral, and dopaminergic abnormalities that resemble schizophrenia. Biol. Psychiatry 69, 432–441. 10.1016/j.biopsych.2010.09.038 21146155

[B84] Ernst M. Pine D. S. Hardin M. (2006). Triadic model of the neurobiology of motivated behavior in adolescence. Psychol. Med. 36, 299–312. 10.1017/S0033291705005891 16472412 PMC2733162

[B85] Fallon J. H. (1988). Topographic organization of ascending dopaminergic projections. Ann. N. Y. Acad. Sci. 537, 1–9. 10.1111/j.1749-6632.1988.tb42093.x 3059916

[B86] Fasano C. Kortleven C. Trudeau L.-E. (2010). Chronic activation of the D2 autoreceptor inhibits both glutamate and dopamine synapse formation and alters the intrinsic properties of mesencephalic dopamine neurons *in vitro*. Eur. J. Neurosci. 32, 1433–1441. 10.1111/j.1460-9568.2010.07397.x 20846243

[B87] Fasano C. Poirier A. DesGroseillers L. Trudeau L.-E. (2008). Chronic activation of the D2 dopamine autoreceptor inhibits synaptogenesis in mesencephalic dopaminergic neurons *in vitro*. Eur. J. Neurosci. 28, 1480–1490. 10.1111/j.1460-9568.2008.06450.x 18973573

[B88] Flores C. Manitt C. Rodaros D. Thompson K. M. Rajabi H. Luk K. C.. (2005). Netrin receptor deficient mice exhibit functional reorganization of dopaminergic systems and do not sensitize to amphetamine. Mol. Psychiatry 10, 606–612. 10.1038/sj.mp.4001607 15534618

[B89] Flores-Barrera E. Thomases D. R. Heng L.-J. Cass D. K. Caballero A. Tseng K. Y. (2014). Late adolescent expression of GluN2B transmission in the prefrontal cortex is input-specific and requires postsynaptic protein kinase A and D1 dopamine receptor signaling. Biol. Psychiatry 75, 508–516. 10.1016/j.biopsych.2013.07.033 24041503 PMC3944379

[B90] Floresco S. B. Jentsch J. D. (2011). Pharmacological enhancement of memory and executive functioning in laboratory animals. Neuropsychopharmacol. Off. Publ. Am. Coll. Neuropsychopharmacol. 36, 227–250. 10.1038/npp.2010.158 20844477 PMC3055518

[B91] Floresco S. B. Magyar O. (2006). Mesocortical dopamine modulation of executive functions: beyond working memory. Psychopharmacology 188, 567–585. 10.1007/s00213-006-0404-5 16670842

[B92] Floresco S. B. West A. R. Ash B. Moore H. Grace A. A. (2003). Afferent modulation of dopamine neuron firing differentially regulates tonic and phasic dopamine transmission. Nat. Neurosci. 6, 968–973. 10.1038/nn1103 12897785

[B93] Fortin D. A. Levine E. S. (2007). Differential effects of endocannabinoids on glutamatergic and GABAergic inputs to layer 5 pyramidal neurons. Cereb. Cortex 17, 163–174. 10.1093/cercor/bhj133 16467564

[B94] Frazier C. R. M. Mason P. Zhuang X. Beeler J. A. (2008). Sucrose exposure in early life alters adult motivation and weight gain. PLoS ONE 3, e3221. 10.1371/journal.pone.0003221 18797507 PMC2529404

[B95] Freund T. F. Katona I. Piomelli D. (2003). Role of endogenous cannabinoids in synaptic signaling. Physiol. Rev. 83, 1017–1066. 10.1152/physrev.00004.2003 12843414

[B96] Friemel C. M. Spanagel R. Schneider M. (2010). Reward sensitivity for a palatable food reward peaks during pubertal developmental in rats. Front. Behav. Neurosci. 4, 39. 10.3389/fnbeh.2010.00039 20700386 PMC2914567

[B97] Fuster J. M. (2001). The prefrontal cortex–an update: time is of the essence. Neuron 30, 319–333. 10.1016/S0896-6273(01)00285-9 11394996

[B98] Gabbott P. L. A. Warner T. A. Jays P. R. L. Salway P. Busby S. J. (2005). Prefrontal cortex in the rat: projections to subcortical autonomic, motor, and limbic centers. J. Comp. Neurol. 492, 145–177. 10.1002/cne.20738 16196030

[B99] Galiñanes G. L. Taravini I. R. E. Murer M. G. (2009). Dopamine-dependent periadolescent maturation of corticostriatal functional connectivity in mouse. J. Neurosci. Off. J. Soc. Neurosci. 29, 2496–2509. 10.1523/JNEUROSCI.4421-08.2009 19244524 PMC2742915

[B100] Gaoni Y. Mechoulam R. (1964). Isolation, structure, and partial synthesis of an active constituent of hashish. J. Am. Chem. Soc. 86, 1646–1647. 10.1021/ja01062a046

[B101] Garris P. A. Wightman R. M. (1994). Different kinetics govern dopaminergic transmission in the amygdala, prefrontal cortex, and striatum: an *in vivo* voltammetric study. J. Neurosci. Off. J. Soc. Neurosci. 14, 442–450. 10.1523/JNEUROSCI.14-01-00442.1994 8283249 PMC6576851

[B102] Gaspar P. Bloch B. Le Moine C. (1995). D1 and D2 receptor gene expression in the rat frontal cortex: cellular localization in different classes of efferent neurons. Eur. J. Neurosci. 7, 1050–1063. 10.1111/j.1460-9568.1995.tb01092.x 7613610

[B103] Ginder D. E. Wright H. R. McLaughlin R. J. (2022). The stoned age: sex differences in the effects of adolescent cannabinoid exposure on prefrontal cortex structure and function in animal models. Int. Rev. Neurobiol. 161, 121–145. 10.1016/bs.irn.2021.07.005 34801167 PMC11290470

[B104] Goldman-Rakic P. S. Muly I. E. C. Williams G. V. (2000). D1 receptors in prefrontal cells and circuits. Brain Res. Rev. 31, 295–301. 10.1016/S0165-0173(99)00045-4 10719156

[B105] González S. Bisogno T. Wenger T. Manzanares J. Milone A. Berrendero F.. (2000). Sex steroid influence on cannabinoid CB(1) receptor mRNA and endocannabinoid levels in the anterior pituitary gland. Biochem. Biophys. Res. Commun. 270, 260–266. 10.1006/bbrc.2000.2406 10733937

[B106] Gorelova N. Seamans J. Yang C. Gorelova N. Seamans J. K. Yang C. R. (2003). Mechanisms of dopamine activation of fast-spiking interneurons that exert inhibition in rat prefrontal cortex. J. Neurophysiol. 88, 3150–3166. 10.1152/jn.00335.2002 12466437

[B107] Grace A. A. Bunney B. S. (1984a). The control of firing pattern in nigral dopamine neurons: burst firing. J. Neurosci. Off. J. Soc. Neurosci. 4, 2877–2890. 10.1523/JNEUROSCI.04-11-02877.1984 6150071 PMC6564720

[B108] Grace A. A. Bunney B. S. (1984b). The control of firing pattern in nigral dopamine neurons: single spike firing. J. Neurosci. Off. J. Soc. Neurosci. 4, 2866–2876. 10.1523/JNEUROSCI.04-11-02866.1984 6150070 PMC6564731

[B109] Grant A. Hoops D. Labelle-Dumais C. Prévost M. Rajabi H. Kolb B.. (2007). Netrin-1 receptor-deficient mice show enhanced mesocortical dopamine transmission and blunted behavioural responses to amphetamine. Eur. J. Neurosci. 26, 3215–3228. 10.1111/j.1460-9568.2007.05888.x 18005074

[B110] Grant A. Speed Z. Labelle-Dumais C. Flores C. (2009). Post-pubertal emergence of a dopamine phenotype in netrin-1 receptor-deficient mice. Eur. J. Neurosci. 30, 1318–1328. 10.1111/j.1460-9568.2009.06919.x 19788579

[B111] Gremel C. M. Chancey J. H. Atwood B. K. Luo G. Neve R. Ramakrishnan C.. (2016). Endocannabinoid modulation of orbitostriatal circuits gates habit formation. Neuron 90, 1312–1324. 10.1016/j.neuron.2016.04.043 27238866 PMC4911264

[B112] Grove J. C. R. Gray L. A. La Santa Medina N. Sivakumar N. Ahn J. S. Corpuz T. V.. (2022). Dopamine subsystems that track internal states. Nature 608, 374–380. 10.1038/s41586-022-04954-0 35831501 PMC9365689

[B113] Gueye A. B. Vendruscolo L. F. de Ávila C. Le Moine C. Darnaudéry M. Cador M. (2018). Unlimited sucrose consumption during adolescence generates a depressive-like phenotype in adulthood. Neuropsychopharmacol. Off. Publ. Am. Coll. Neuropsychopharmacol. 43, 2627–2635. 10.1038/s41386-018-0025-9 29487370 PMC6224580

[B114] Haber S. N. (2003). The primate basal ganglia: parallel and integrative networks. J. Chem. Neuroanat. 26, 317–330. 10.1016/j.jchemneu.2003.10.003 14729134

[B115] Hajnal A. Norgren R. (2001). Accumbens dopamine mechanisms in sucrose intake. Brain Res. 904, 76–84. 10.1016/S0006-8993(01)02451-9 11516413

[B116] Hajnal A. Smith G. P. Norgren R. (2004). Oral sucrose stimulation increases accumbens dopamine in the rat. Am. J. Physiol. Regul. Integr. Comp. Physiol. 286, R31–37. 10.1152/ajpregu.00282.2003 12933362

[B117] Hammerslag L. R. Gulley J. M. (2016). Sex differences in behavior and neural development and their role in adolescent vulnerability to substance use. Behav. Brain Res. 298, 15–26. 10.1016/j.bbr.2015.04.008 25882721 PMC4603997

[B118] Häring M. Guggenhuber S. Lutz B. (2012). Neuronal populations mediating the effects of endocannabinoids on stress and emotionality. Neuroscience 204, 145–158. 10.1016/j.neuroscience.2011.12.035 22233782

[B119] Harkany T. Guzmán M. Galve-Roperh I. Berghuis P. Devi L. A. Mackie K. (2007). The emerging functions of endocannabinoid signaling during CNS development. Trends Pharmacol. Sci. 28, 83–92. 10.1016/j.tips.2006.12.004 17222464

[B120] Harkany T. Keimpema E. Barabás K. Mulder J. (2008). Endocannabinoid functions controlling neuronal specification during brain development. Mol. Cell. Endocrinol. 286, S84–90. 10.1016/j.mce.2008.02.011 18394789

[B121] Heidbreder C. A. Groenewegen H. J. (2003). The medial prefrontal cortex in the rat: evidence for a dorso-ventral distinction based upon functional and anatomical characteristics. Neurosci. Biobehav. Rev. 27, 555–579. 10.1016/j.neubiorev.2003.09.003 14599436

[B122] Heng L. Beverley J. A. Steiner H. Tseng K. Y. (2011a). Differential developmental trajectories for CB1 cannabinoid receptor expression in limbic/associative and sensorimotor cortical areas. Synap. N. Y. N. 65, 278–286. 10.1002/syn.20844 20687106 PMC2978763

[B123] Heng L. Markham J. A. Hu X.-T. Tseng K. Y. (2011b). Concurrent upregulation of postsynaptic L-type Ca^2+^ channel function and protein kinase A signaling is required for the periadolescent facilitation of Ca^2+^ plateau potentials and dopamine D1 receptor modulation in the prefrontal cortex. Neuropharmacology 60, 953–962. 10.1016/j.neuropharm.2011.01.041 21288471 PMC3077898

[B124] Herkenham M. Lynn A. B. Little M. D. Johnson M. R. Melvin L. S. de Costa B. R.. (1990). Cannabinoid receptor localization in brain. Proc. Natl. Acad. Sci. U. S. A. 87, 1932–1936. 10.1073/pnas.87.5.1932 2308954 PMC53598

[B125] Hernandez L. Hoebel B. G. (1990). Feeding can enhance dopamine turnover in the prefrontal cortex. Brain Res. Bull. 25, 975–979. 10.1016/0361-9230(90)90197-8 2289179

[B126] Higuera-Matas A. Soto-Montenegro M. L. del Olmo N. Miguéns M. Torres I. Vaquero J. J.. (2008). Augmented acquisition of cocaine self-administration and altered brain glucose metabolism in adult female but not male rats exposed to a cannabinoid agonist during adolescence. Neuropsychopharmacol. Off. Publ. Am. Coll. Neuropsychopharmacol. 33, 806–813. 10.1038/sj.npp.1301467 17551541

[B127] Hill M. N. McLaughlin R. J. Pan B. Fitzgerald M. L. Roberts C. J. Lee T. T.-Y.. (2011). Recruitment of prefrontal cortical endocannabinoid signaling by glucocorticoids contributes to termination of the stress response. J. Neurosci. Off. J. Soc. Neurosci. 31, 10506–10515. 10.1523/JNEUROSCI.0496-11.2011 21775596 PMC3179266

[B128] Hill M. N. Tasker J. G. (2012). Endocannabinoid signaling, glucocorticoid-mediated negative feedback, and regulation of the hypothalamic-pituitary-adrenal axis. Neuroscience 204, 5–16. 10.1016/j.neuroscience.2011.12.030 22214537 PMC3288468

[B129] Hillard C. J. Wilkison D. M. Edgemond W. S. Campbell W. B. (1995). Characterization of the kinetics and distribution of N-arachidonylethanolamine (anandamide) hydrolysis by rat brain. Biochim. Biophys. Acta 1257, 249–256. 10.1016/0005-2760(95)00087-S 7647100

[B130] Hoops D. Flores C. (2017). Making dopamine connections in adolescence. Trends Neurosci. 40, 709–719. 10.1016/j.tins.2017.09.004 29032842 PMC5705341

[B131] Hoover W. B. Vertes R. P. (2007). Anatomical analysis of afferent projections to the medial prefrontal cortex in the rat. Brain Struct. Funct. 212, 149–179. 10.1007/s00429-007-0150-4 17717690

[B132] Howlett A. C. (2002). The cannabinoid receptors. Prostaglandins Other Lipid Mediat. 68–69, 619–631. 10.1016/S0090-6980(02)00060-6 12432948

[B133] Howlett A. C. Mukhopadhyay S. (2000). Cellular signal transduction by anandamide and 2-arachidonoylglycerol. Chem. Phys. Lipids 108, 53–70. 10.1016/S0009-3084(00)00187-0 11106782

[B134] Huestis M. A. Gorelick D. A. Heishman S. J. Preston K. L. Nelson R. A. Moolchan E. T.. (2001). Blockade of effects of smoked marijuana by the CB1-selective cannabinoid receptor antagonist SR141716. Arch. Gen. Psychiatry 58, 322–328. 10.1001/archpsyc.58.4.322 11296091

[B135] Islam K. U. S. Meli N. Blaess S. (2021). The development of the mesoprefrontal dopaminergic system in health and disease. Front. Neural Circuits 15, 746582. 10.3389/fncir.2021.746582 34712123 PMC8546303

[B136] Johnson C. M. Loucks F. A. Peckler H. Thomas A. W. Janak P. H. Wilbrecht L. (2016). Long-range orbitofrontal and amygdala axons show divergent patterns of maturation in the frontal cortex across adolescence. Dev. Cogn. Neurosci. 18, 113–120. 10.1016/j.dcn.2016.01.005 26896859 PMC5283395

[B137] Julian M. D. Martin A. B. Cuellar B. Rodriguez De Fonseca F. Navarro M. Moratalla R.. (2003). Neuroanatomical relationship between type 1 cannabinoid receptors and dopaminergic systems in the rat basal ganglia. Neuroscience 119, 309–318. 10.1016/S0306-4522(03)00070-8 12763090

[B138] Kalsbeek A. Voorn P. Buijs R. M. Pool C. W. Uylings H. B. (1988). Development of the dopaminergic innervation in the prefrontal cortex of the rat. J. Comp. Neurol. 269, 58–72. 10.1002/cne.902690105 3361004

[B139] Kano M. Ohno-Shosaku T. Hashimotodani Y. Uchigashima M. Watanabe M. (2009). Endocannabinoid-mediated control of synaptic transmission. Physiol. Rev. 89, 309–380. 10.1152/physrev.00019.2008 19126760

[B140] Karreman M. Moghaddam B. (1996). The prefrontal cortex regulates the basal release of dopamine in the limbic striatum: an effect mediated by ventral tegmental area. J. Neurochem. 66, 589–598. 10.1046/j.1471-4159.1996.66020589.x 8592128

[B141] Kendig M. D. (2014). Cognitive and behavioural effects of sugar consumption in rodents. A review. Appetite 80, 41–54. 10.1016/j.appet.2014.04.028 24816323

[B142] Kendig M. D. Boakes R. A. Rooney K. B. Corbit L. H. (2013). Chronic restricted access to 10% sucrose solution in adolescent and young adult rats impairs spatial memory and alters sensitivity to outcome devaluation. Physiol. Behav. 120, 164–172. 10.1016/j.physbeh.2013.08.012 23954407

[B143] Kenny P. J. (2011). Common cellular and molecular mechanisms in obesity and drug addiction. Nat. Rev. Neurosci. 12, 638–651. 10.1038/nrn3105 22011680

[B144] Kettenmann H. Kirchhoff F. Verkhratsky A. (2013). Microglia: new roles for the synaptic stripper. Neuron 77, 10–18. 10.1016/j.neuron.2012.12.023 23312512

[B145] Kim Y. Simon N. W. Wood J. Moghaddam B. (2016). Reward anticipation is encoded differently by adolescent ventral tegmental area neurons. Biol. Psychiatry 79, 878–886. 10.1016/j.biopsych.2015.04.026 26067679 PMC4636980

[B146] Kiritoshi T. Sun H. Ren W. Stauffer S. R. Lindsley C. W. Conn P. J.. (2013). Modulation of pyramidal cell output in the medial prefrontal cortex by mGluR5 interacting with CB1. Neuropharmacology 66, 170–178. 10.1016/j.neuropharm.2012.03.024 22521499 PMC3568505

[B147] Kolk S. M. Rakic P. (2022). Development of prefrontal cortex. Neuropsychopharmacology 47, 41–57. 10.1038/s41386-021-01137-9 34645980 PMC8511863

[B148] Kopec A. M. Smith C. J. Ayre N. R. Sweat S. C. Bilbo S. D. (2018). Microglial dopamine receptor elimination defines sex-specific nucleus accumbens development and social behavior in adolescent rats. Nat. Commun. 9, 3769. 10.1038/s41467-018-06118-z 30254300 PMC6156594

[B149] Kreitzer A. C. Regehr W. G. (2002). Retrograde signaling by endocannabinoids. Curr. Opin. Neurobiol. 12, 324–330. 10.1016/S0959-4388(02)00328-8 12049940

[B150] Kritzer M. F. (1998). Perinatal gonadectomy exerts regionally selective, lateralized effects on the density of axons immunoreactive for tyrosine hydroxylase in the cerebral cortex of adult male rats. J. Neurosci. Off. J. Soc. Neurosci. 18, 10735–10748. 10.1523/JNEUROSCI.18-24-10735.1998 9852608 PMC6793338

[B151] Kritzer M. F. Adler A. Marotta J. Smirlis T. (1999). Regionally selective effects of gonadectomy on cortical catecholamine innervation in adult male rats are most disruptive to afferents in prefrontal cortex. Cereb. Cortex N. Y. N. 9, 507–518. 10.1093/cercor/9.5.507 10450895

[B152] Kritzer M. F. Creutz L. M. (2008). Region and sex differences in constituent dopamine neurons and immunoreactivity for intracellular estrogen and androgen receptors in mesocortical projections in rats. J. Neurosci. 28, 9525–9535. 10.1523/JNEUROSCI.2637-08.2008 18799684 PMC2613180

[B153] Labouèbe G. Liu S. Dias C. Zou H. Wong J. C. Y. Karunakaran S.. (2013). Insulin induces long-term depression of ventral tegmental area dopamine neurons *via* endocannabinoids. Nat. Neurosci. 16, 300–308. 10.1038/nn.3321 23354329 PMC4072656

[B154] Labouesse M. A. Cola R. B. Patriarchi T. (2020). GPCR-based dopamine sensors-a detailed guide to inform sensor choice for *in vivo* imaging. Int. J. Mol. Sci. 21, E8048. 10.3390/ijms21218048 33126757 PMC7672611

[B155] Labouesse M. A. Lassalle O. Richetto J. Iafrati J. Weber-Stadlbauer U. Notter T.. (2017). Hypervulnerability of the adolescent prefrontal cortex to nutritional stress *via* reelin deficiency. Mol. Psychiatry 22, 961–971. 10.1038/mp.2016.193 27843148

[B156] Lafourcade M. Elezgarai I. Mato S. Bakiri Y. Grandes P. Manzoni O. J. (2007). Molecular components and functions of the endocannabinoid system in mouse prefrontal cortex. PLoS ONE 2, e709. 10.1371/journal.pone.0000709 17684555 PMC1933592

[B157] Lambe E. K. Krimer L. S. Goldman-Rakic P. S. (2000). Differential postnatal development of catecholamine and serotonin inputs to identified neurons in prefrontal cortex of rhesus monkey. J. Neurosci. 20, 8780–8787. 10.1523/JNEUROSCI.20-23-08780.2000 11102486 PMC6773090

[B158] Lammel S. Hetzel A. Häckel O. Jones I. Liss B. Roeper J. (2008). Unique properties of mesoprefrontal neurons within a dual mesocorticolimbic dopamine system. Neuron 57, 760–773. 10.1016/j.neuron.2008.01.022 18341995

[B159] Lammel S. Lim B. K. Malenka R. C. (2014). Reward and aversion in a heterogeneous midbrain dopamine system. Neuropharmacology 76, 351–359. 10.1016/j.neuropharm.2013.03.019 23578393 PMC3778102

[B160] Lapish C. C. Kroener S. Durstewitz D. Lavin A. Seamans J. K. (2007). The ability of the mesocortical dopamine system to operate in distinct temporal modes. Psychopharmacology 191, 609–625. 10.1007/s00213-006-0527-8 17086392 PMC5509053

[B161] Lau B. K. Cota D. Cristino L. Borgland S. L. (2017). Endocannabinoid modulation of homeostatic and non-homeostatic feeding circuits. Neuropharmacology 124, 38–51. 10.1016/j.neuropharm.2017.05.033 28579186

[B162] Laubach M. Amarante L. M. Swanson K. White S. R. (2018). What, if anything, is rodent prefrontal cortex? eNeuro 5, ENEURO.0315-18.2018. 10.1523/ENEURO.0315-18.2018 30406193 PMC6220587

[B163] Laviolette S. R. Grace A. A. (2006). The roles of cannabinoid and dopamine receptor systems in neural emotional learning circuits: implications for schizophrenia and addiction. Cell. Mol. Life Sci. CMLS 63, 1597–1613. 10.1007/s00018-006-6027-5 16699809 PMC11136137

[B164] Le Moine C. Gaspar P. (1998). Subpopulations of cortical GABAergic interneurons differ by their expression of D1 and D2 dopamine receptor subtypes. Brain Res. Mol. Brain Res. 58, 231–236. 10.1016/S0169-328X(98)00118-1 9685656

[B165] Lenroot R. K. Giedd J. N. (2006). Brain development in children and adolescents: insights from anatomical magnetic resonance imaging. Neurosci. Biobehav. Rev. 30, 718–729. 10.1016/j.neubiorev.2006.06.001 16887188

[B166] Leslie C. A. Robertson M. W. Cutler A. J. Bennett J. P. (1991). Postnatal development of D1 dopamine receptors in the medial prefrontal cortex, striatum and nucleus accumbens of normal and neonatal 6-hydroxydopamine treated rats: a quantitative autoradiographic analysis. Brain Res. Dev. Brain Res. 62, 109–114. 10.1016/0165-3806(91)90195-O 1836980

[B167] Levitt P. Moore R. Y. (1979). Development of the noradrenergic innervation of neocortex. Brain Res. 162, 243–259. 10.1016/0006-8993(79)90287-7 761089

[B168] Lewis D. A. González-Burgos G. (2008). Neuroplasticity of neocortical circuits in schizophrenia. Neuropsychopharmacol. Off. Publ. Am. Coll. Neuropsychopharmacol. 33, 141–165. 10.1038/sj.npp.1301563 17805309

[B169] Lidov H. G. Grzanna R. Molliver M. E. (1980). The serotonin innervation of the cerebral cortex in the rat–an immunohistochemical analysis. Neuroscience 5, 207–227. 10.1016/0306-4522(80)90099-8 6990293

[B170] Liu S. Borgland S. L. (2015). Regulation of the mesolimbic dopamine circuit by feeding peptides. Neuroscience 289, 19–42. 10.1016/j.neuroscience.2014.12.046 25583635

[B171] Liu S. Globa A. K. Mills F. Naef L. Qiao M. Bamji S. X.. (2016). Consumption of palatable food primes food approach behavior by rapidly increasing synaptic density in the VTA. Proc. Natl. Acad. Sci. U. S. A. 113, 2520–2525. 10.1073/pnas.1515724113 26884159 PMC4780604

[B172] Long L. E. Lind J. Webster M. Weickert C. S. (2012). Developmental trajectory of the endocannabinoid system in human dorsolateral prefrontal cortex. BMC Neurosci. 13, 87. 10.1186/1471-2202-13-87 22827915 PMC3464170

[B173] Lovelace J. W. Corches A. Vieira P. A. Mackie K. Korzus E. (2015). An animal model of female adolescent cannabinoid exposure elicits a long-lasting deficit in presynaptic long-term plasticity. Neuropharmacology 99, 242–255. 10.1016/j.neuropharm.2015.04.034 25979486 PMC4644105

[B174] Lovinger D. M. (2008). Presynaptic modulation by endocannabinoids. Handb. Exp. Pharmacol. 14, 435–477. 10.1007/978-3-540-74805-2_14 18064422

[B175] Lowe C. J. Reichelt A. C. Hall P. A. (2019). The prefrontal cortex and obesity: a health neuroscience perspective. Trends Cogn. Sci. 23, 349–361. 10.1016/j.tics.2019.01.005 30824229

[B176] Lu H.-C. Mackie K. (2016). An introduction to the endogenous cannabinoid system. Biol. Psychiatry 79, 516–525. 10.1016/j.biopsych.2015.07.028 26698193 PMC4789136

[B177] Maccarrone M. (2020). Missing pieces to the endocannabinoid puzzle. Trends Mol. Med. 26, 263–272. 10.1016/j.molmed.2019.11.002 31822395

[B178] Maccarrone M. Gasperi V. Catani M. V. Diep T. A. Dainese E. Hansen H. S.. (2010). The endocannabinoid system and its relevance for nutrition. Annu. Rev. Nutr. 30, 423–440. 10.1146/annurev.nutr.012809.104701 20645854

[B179] Maccarrone M. Guzmán M. Mackie K. Doherty P. Harkany T. (2014). Programming of neural cells by (endo)cannabinoids: from physiological rules to emerging therapies. Nat. Rev. Neurosci. 15, 786–801. 10.1038/nrn3846 25409697 PMC4765324

[B180] Mackie K. (2006). Mechanisms of CB1 receptor signaling: endocannabinoid modulation of synaptic strength. Int. J. Obes. 30, S19–S23. 10.1038/sj.ijo.0803273 16570100

[B181] Mahler S. V. Smith K. S. Berridge K. C. (2007). Endocannabinoid hedonic hotspot for sensory pleasure: anandamide in nucleus accumbens shell enhances “liking” of a sweet reward. Neuropsychopharmacol. Off. Publ. Am. Coll. Neuropsychopharmacol. 32, 2267–2278. 10.1038/sj.npp.1301376 17406653

[B182] Mallya A. P. Wang H.-D. Lee H. N. R. Deutch A. Y. (2019). Microglial pruning of synapses in the prefrontal cortex during adolescence. Cereb. Cortex N. Y. N. 29, 1634–1643. 10.1093/cercor/bhy061 29668872 PMC6418387

[B183] Manitt C. Eng C. Pokinko M. Ryan R. T. Torres-Berrío A. Lopez J. P.. (2013). DCC orchestrates the development of the prefrontal cortex during adolescence and is altered in psychiatric patients. Transl. Psychiatry 3, e338. 10.1038/tp.2013.105 24346136 PMC4030324

[B184] Manitt C. Labelle-Dumais C. Eng C. Grant A. Mimee A. Stroh T.. (2010). Peri-pubertal emergence of UNC-5 homologue expression by dopamine neurons in rodents. PLoS ONE 5, e11463. 10.1371/journal.pone.0011463 20628609 PMC2900213

[B185] Manitt C. Mimee A. Eng C. Pokinko M. Stroh T. Cooper H. M.. (2011). The netrin receptor DCC is required in the pubertal organization of mesocortical dopamine circuitry. J. Neurosci. Off. J. Soc. Neurosci. 31, 8381–8394. 10.1523/JNEUROSCI.0606-11.2011 21653843 PMC6623341

[B186] Mantz J. Thierry A. M. Glowinski J. (1989). Effect of noxious tail pinch on the discharge rate of mesocortical and mesolimbic dopamine neurons: selective activation of the mesocortical system. Brain Res. 476, 377–381. 10.1016/0006-8993(89)91263-8 2702475

[B187] Marinelli M. McCutcheon J. E. (2014). Heterogeneity of dopamine neuron activity across traits and states. Neuroscience 282, 176–197. 10.1016/j.neuroscience.2014.07.034 25084048 PMC4312268

[B188] Marshall A. T. Liu A. T. Murphy N. P. Maidment N. T. Ostlund S. B. (2017). Sex-specific enhancement of palatability-driven feeding in adolescent rats. PLoS ONE 12, e0180907. 10.1371/journal.pone.0180907 28708901 PMC5510835

[B189] Marshall A. T. Munson C. N. Maidment N. T. Ostlund S. B. (2020). Reward-predictive cues elicit excessive reward seeking in adolescent rats. Dev. Cogn. Neurosci. 45, 100838. 10.1016/j.dcn.2020.100838 32846387 PMC7451619

[B190] Marsicano G. Lutz B. (1999). Expression of the cannabinoid receptor CB1 in distinct neuronal subpopulations in the adult mouse forebrain. Eur. J. Neurosci. 11, 4213–4225. 10.1046/j.1460-9568.1999.00847.x 10594647

[B191] Mastwal S. Ye Y. Ren M. Jimenez D. V. Martinowich K. Gerfen C. R.. (2014). Phasic dopamine neuron activity elicits unique mesofrontal plasticity in adolescence. J. Neurosci. 34, 9484–9496. 10.1523/JNEUROSCI.1114-14.2014 25031392 PMC4099535

[B192] Mateo Y. Johnson K. A. Covey D. P. Atwood B. K. Wang H.-L. Zhang S.. (2017). Endocannabinoid actions on cortical terminals orchestrate local modulation of dopamine release in the nucleus accumbens. Neuron 96, 1112–1126.e5. 10.1016/j.neuron.2017.11.012 29216450 PMC5728656

[B193] Matthews M. Bondi C. Torres G. Moghaddam B. (2013). Reduced presynaptic dopamine activity in adolescent dorsal striatum. Neuropsychopharmacol. Off. Publ. Am. Coll. Neuropsychopharmacol. 38, 1344–1351. 10.1038/npp.2013.32 23358239 PMC3656377

[B194] McCane A. M. Wegener M. A. Faraji M. Rivera-Garcia M. T. Wallin-Miller K. G. Costa V. D.. (2021). Adolescent dopamine neurons represent reward differently during action and state guided learning. J. Neurosci. Off. J. Soc. Neurosci. 41, 9419–9430. 10.1523/JNEUROSCI.1321-21.2021 34611024 PMC8580150

[B195] McCutcheon J. E. (2015). The role of dopamine in the pursuit of nutritional value. Physiol. Behav. 152, 408–415. 10.1016/j.physbeh.2015.05.003 25957911

[B196] McCutcheon J. E. Conrad K. L. Carr S. B. Ford K. A. McGehee D. S. Marinelli M. (2012). Dopamine neurons in the ventral tegmental area fire faster in adolescent rats than in adults. J. Neurophysiol. 108, 1620–1630. 10.1152/jn.00077.2012 22723669 PMC3544953

[B197] McCutcheon J. E. Marinelli M. (2009). Age matters. Eur. J. Neurosci. 29, 997–1014. 10.1111/j.1460-9568.2009.06648.x 19291226 PMC2761206

[B198] McLaughlin R. J. Hill M. N. Gorzalka B. B. (2014). A critical role for prefrontocortical endocannabinoid signaling in the regulation of stress and emotional behavior. Neurosci. Biobehav. Rev. 42, 116–131. 10.1016/j.neubiorev.2014.02.006 24582908

[B199] Mechoulam R. Ben-Shabat S. Hanus L. Ligumsky M. Kaminski N. E. Schatz A. R.. (1995). Identification of an endogenous 2-monoglyceride, present in canine gut, that binds to cannabinoid receptors. Biochem. Pharmacol. 50, 83–90. 10.1016/0006-2952(95)00109-D 7605349

[B200] Melis M. Pistis M. Perra S. Muntoni A. L. Pillolla G. Gessa G. L. (2004). Endocannabinoids mediate presynaptic inhibition of glutamatergic transmission in rat ventral tegmental area dopamine neurons through activation of CB1 receptors. J. Neurosci. Off. J. Soc. Neurosci. 24, 53–62. 10.1523/JNEUROSCI.4503-03.2004 14715937 PMC6729571

[B201] Meyer H. C. Lee F. S. Gee D. G. (2018). The role of the endocannabinoid system and genetic variation in adolescent brain development. Neuropsychopharmacology 43, 21–33. 10.1038/npp.2017.143 28685756 PMC5719094

[B202] Miech R. A. Patrick M. E. O'Malley P. M. Johnston L. D. Bachman J. G. (2020). Trends in reported marijuana vaping among US adolescents, 2017–2019. J. Am. Med. Assoc. 323, 475–476. 10.1001/jama.2019.20185 31848566 PMC6990699

[B203] Miller E. K. Cohen J. D. (2001). An integrative theory of prefrontal cortex function. Annu. Rev. Neurosci. 24, 167–202. 10.1146/annurev.neuro.24.1.167 11283309

[B204] Montague P. R. Hyman S. E. Cohen J. D. (2004). Computational roles for dopamine in behavioural control. Nature 431, 760–767. 10.1038/nature03015 15483596

[B205] Morales M. Margolis E. B. (2017). Ventral tegmental area: cellular heterogeneity, connectivity and behaviour. Nat. Rev. Neurosci. 18, 73–85. 10.1038/nrn.2016.165 28053327

[B206] Mutlu A. K. Schneider M. Debban,é M. Badoud D. Eliez S. Schaer M. (2013). Sex differences in thickness, and folding developments throughout the cortex. NeuroImage 82, 200–207. 10.1016/j.neuroimage.2013.05.076 23721724

[B207] Nakazawa K. Zsiros V. Jiang Z. Nakao K. Kolata S. Zhang S.. (2012). GABAergic interneuron origin of schizophrenia pathophysiology. Neuropharmacology 62, 1574–1583. 10.1016/j.neuropharm.2011.01.022 21277876 PMC3090452

[B208] Naneix F. Darlot F. Coutureau E. Cador M. (2016). Long-lasting deficits in hedonic and nucleus accumbens reactivity to sweet rewards by sugar overconsumption during adolescence. Eur. J. Neurosci. 43, 671–680. 10.1111/ejn.13149 26762310

[B209] Naneix F. Darlot F. De Smedt-Peyrusse V. Pape J.-R. Coutureau E. Cador M. (2018). Protracted motivational dopamine-related deficits following adolescence sugar overconsumption. Neuropharmacology 129, 16–25. 10.1016/j.neuropharm.2017.11.021 29146502

[B210] Naneix F. Marchand A. R. Di Scala G. Pape J.-R. Coutureau E. (2012). Parallel maturation of goal-directed behavior and dopaminergic systems during adolescence. J. Neurosci. Off. J. Soc. Neurosci. 32, 16223–16232. 10.1523/JNEUROSCI.3080-12.2012 23152606 PMC6794026

[B211] Naneix F. Marchand A. R. Pichon A. Pape J.-R. Coutureau E. (2013). Adolescent stimulation of D2 receptors alters the maturation of dopamine-dependent goal-directed behavior. Neuropsychopharmacol. Off. Publ. Am. Coll. Neuropsychopharmacol. 38, 1566–1574. 10.1038/npp.2013.55 23443719 PMC3682151

[B212] Naneix F. Tantot F. Glangetas C. Kaufling J. Janthakhin Y. Boitard C.. (2017). Impact of early consumption of high-fat diet on the mesolimbic dopaminergic system. eNeuro 4, ENEURO.0120-17.2017. 10.1523/ENEURO.0120-17.2017 28580417 PMC5454405

[B213] Niv Y. (2007). Cost, benefit, tonic, phasic: what do response rates tell us about dopamine and motivation? Ann. N. Y. Acad. Sci. 1104, 357–376. 10.1196/annals.1390.018 17416928

[B214] Nolan S. O. Zachry J. E. Johnson A. R. Brady L. J. Siciliano C. A. Calipari E. S. (2020). Direct dopamine terminal regulation by local striatal microcircuitry. J. Neurochem. 155, 475–493. 10.1111/jnc.15034 32356315 PMC7606645

[B215] Nomura Y. Naitoh F. Segawa T. (1976). Regional changes in monoamine content and uptake of the rat brain during postnatal development. Brain Res. 101, 305–315. 10.1016/0006-8993(76)90271-7 1244975

[B216] O'Carroll R. (2000). Cognitive impairment in schizophrenia. Adv. Psychiatr. Treat. 6, 161–168. 10.1192/apt.6.3.161

[B217] O'Donnell P. (2011). Adolescent onset of cortical disinhibition in schizophrenia: insights from animal models. Schizophr. Bull. 37, 484–492. 10.1093/schbul/sbr028 21505115 PMC3080677

[B218] Ogden C. L. Carroll M. D. Lawman H. G. Fryar C. D. Kruszon-Moran D. Kit B. K.. (2016). Trends in obesity prevalence among children and adolescents in the United States, 1988–1994 through 2013–2014. J. Am. Med. Assoc. 315, 2292–2299. 10.1001/jama.2016.6361 27272581 PMC6361521

[B219] Oleson E. B. Beckert M. V. Morra J. T. Lansink C. S. Cachope R. Abdullah R. A.. (2012). Endocannabinoids shape accumbal encoding of cue-motivated behavior *via* CB1 receptor activation in the ventral tegmentum. Neuron 73, 360–373. 10.1016/j.neuron.2011.11.018 22284189 PMC3269037

[B220] O'Shea M. Singh M. E. McGregor I. S. Mallet P. E. (2004). Chronic cannabinoid exposure produces lasting memory impairment and increased anxiety in adolescent but not adult rats. J. Psychopharmacol. Oxf. Engl. 18, 502–508. 10.1177/026988110401800407 15582916

[B221] Parish C. L. Finkelstein D. I. Drago J. Borrelli E. Horne M. K. (2001). The role of dopamine receptors in regulating the size of axonal arbors. J. Neurosci. 21, 5147–5157. 10.1523/JNEUROSCI.21-14-05147.2001 11438590 PMC6762846

[B222] Pattij T. Wiskerke J. Schoffelmeer A. N. M. (2008). Cannabinoid modulation of executive functions. Eur. J. Pharmacol. 585, 458–463. 10.1016/j.ejphar.2008.02.099 18423599

[B223] Paulus M. P. (2007). Decision-making dysfunctions in psychiatry–altered homeostatic processing? Science 318, 602–606. 10.1126/science.1142997 17962553

[B224] Paus T. Keshavan M. Giedd J. N. (2008). Why do many psychiatric disorders emerge during adolescence? Nat. Rev. Neurosci. 9, 947–957. 10.1038/nrn2513 19002191 PMC2762785

[B225] Peters K. Z. Cheer J. F. Tonini R. (2021a). Modulating the neuromodulators: dopamine, serotonin, and the endocannabinoid system. Trends Neurosci. 44, 464–477. 10.1016/j.tins.2021.02.001 33674134 PMC8159866

[B226] Peters K. Z. Oleson E. B. Cheer J. F. (2021b). A brain on cannabinoids: the role of dopamine release in reward seeking and addiction. Cold Spring Harb. Perspect. Med. 11, a039305. 10.1101/cshperspect.a039305 31964646 PMC7778214

[B227] Peters K. Z. Zlebnik N. E. Cheer J. F. (2021c). Chapter Three - Cannabis exposure during adolescence: a uniquely sensitive period for neurobiological effects, in International Review of Neurobiology Effects of Peri-Adolescent Licit and Illicit Drug Use on the Developing CNS Part II, eds Bell R. L. Rahman S. (Cambridge, MA: Academic Press), 95–120. 10.1016/bs.irn.2021.07.002 34801175

[B228] Piscitelli F. Di Marzo V. (2012). “Redundancy” of endocannabinoid inactivation: new challenges and opportunities for pain control. ACS Chem. Neurosci. 3, 356–363. 10.1021/cn300015x 22860203 PMC3382450

[B229] Pistis M. Ferraro L. Pira L. Flore G. Tanganelli S. Gessa G. L.. (2002). Δ^9^-Tetrahydrocannabinol decreases extracellular GABA and increases extracellular glutamate and dopamine levels in the rat prefrontal cortex: an *in vivo* microdialysis study. Brain Res. 948, 155–158. 10.1016/S0006-8993(02)03055-X 12383968

[B230] Pitts E. G. Stowe T. A. Christensen B. A. Ferris M. J. (2020). Comparing dopamine release, uptake, and D2 autoreceptor function across the ventromedial to dorsolateral striatum in adolescent and adult male and female rats. Neuropharmacology 175, 108163. 10.1016/j.neuropharm.2020.108163 32479812 PMC7513623

[B231] Privitera G. J. Zavala A. R. Sanabria F. Sotak K. L. (2011). High fat diet intake during pre and periadolescence impairs learning of a conditioned place preference in adulthood. Behav. Brain Funct. 7, 21. 10.1186/1744-9081-7-21 21703027 PMC3146828

[B232] Rabasa C. Winsa-Jörnulf J. Vogel H. Babaei C. S. Askevik K. Dickson S. L. (2016). Behavioral consequences of exposure to a high fat diet during the post-weaning period in rats. Horm. Behav. 85, 56–66. 10.1016/j.yhbeh.2016.07.008 27487416

[B233] Rangel A. Camerer C. Montague P. R. (2008). A framework for studying the neurobiology of value-based decision making. Nat. Rev. Neurosci. 9, 545–556. 10.1038/nrn2357 18545266 PMC4332708

[B234] Raznahan A. Shaw P. W. Lerch J. P. Clasen L. S. Greenstein D. Berman R.. (2014). Longitudinal four-dimensional mapping of subcortical anatomy in human development. Proc. Natl. Acad. Sci. U. S. A. 111, 1592–1597. 10.1073/pnas.1316911111 24474784 PMC3910572

[B235] Reichelt A. C. (2016). Adolescent maturational transitions in the prefrontal cortex and dopamine signaling as a risk factor for the development of obesity and high fat/high sugar diet induced cognitive deficits. Front. Behav. Neurosci. 10, 189. 10.3389/fnbeh.2016.00189 27790098 PMC5061823

[B236] Reichelt A. C. Abbott K. N. Westbrook R. F. Morris M. J. (2016). Differential motivational profiles following adolescent sucrose access in male and female rats. Physiol. Behav. 157, 13–19. 10.1016/j.physbeh.2016.01.038 26826605

[B237] Reichelt A. C. Gibson G. D. Abbott K. N. Hare D. J. (2019). A high-fat high-sugar diet in adolescent rats impairs social memory and alters chemical markers characteristic of atypical neuroplasticity and parvalbumin interneuron depletion in the medial prefrontal cortex. Food Funct. 10, 1985–1998. 10.1039/C8FO02118J 30900711

[B238] Reichelt A. C. Killcross S. Hambly L. D. Morris M. J. Westbrook R. F. (2015). Impact of adolescent sucrose access on cognitive control, recognition memory, and parvalbumin immunoreactivity. Learn. Mem. Cold Spring Harb. N 22, 215–224. 10.1101/lm.038000.114 25776039 PMC4371171

[B239] Reichelt A. C. Rank M. M. (2017). The impact of junk foods on the adolescent brain. Birth Defects Res. 109, 1649–1658. 10.1002/bdr2.1173 29251841

[B240] Renard J. Krebs M.-O. Jay T. M. Le Pen G. (2013). Long-term cognitive impairments induced by chronic cannabinoid exposure during adolescence in rats: a strain comparison. Psychopharmacology 225, 781–790. 10.1007/s00213-012-2865-z 22983145

[B241] Renard J. Rushlow W. J. Laviolette S. R. (2016). What can rats tell us about adolescent cannabis exposure? Insights from preclinical research. Can. J. Psychiatry Rev. Can. Psychiatr. 61, 328–334. 10.1177/0706743716645288 27254841 PMC4872245

[B242] Renard J. Rushlow W. J. Laviolette S. R. (2018). Effects of adolescent THC exposure on the prefrontal GABAergic system: implications for schizophrenia-related psychopathology. Front. Psychiatry 9, 281. 10.3389/fpsyt.2018.00281 30013490 PMC6036125

[B243] Renard J. Szkudlarek H. J. Kramar C. P. Jobson C. E. L. Moura K. Rushlow W. J.. (2017). Adolescent THC exposure causes enduring prefrontal cortical disruption of GABAergic inhibition and dysregulation of sub-cortical dopamine function. Sci. Rep. 7, 11420. 10.1038/s41598-017-11645-8 28900286 PMC5595795

[B244] Reynolds L. M. Flores C. (2021). Mesocorticolimbic dopamine pathways across adolescence: diversity in development. Front. Neural Circuits 15, 735625. 10.3389/fncir.2021.735625 34566584 PMC8456011

[B245] Reynolds L. M. Makowski C. S. Yogendran S. V. Kiessling S. Cermakian N. Flores C. (2015). Amphetamine in adolescence disrupts the development of medial prefrontal cortex dopamine connectivity in a DCC-dependent manner. Neuropsychopharmacology 40, 1101–1112. 10.1038/npp.2014.287 25336209 PMC4367452

[B246] Reynolds L. M. Pokinko M. Torres-Berrío A. Cuesta S. Lambert L. C. Del Cid Pellitero E.. (2018). DCC receptors drive prefrontal cortex maturation by determining dopamine axon targeting in adolescence. Biol. Psychiatry 83, 181–192. 10.1016/j.biopsych.2017.06.009 28720317 PMC5723533

[B247] Rice M. E. Patel J. C. Cragg S. J. (2011). Dopamine release in the basal ganglia. Neuroscience 198, 112–137. 10.1016/j.neuroscience.2011.08.066 21939738 PMC3357127

[B248] Riddle R. Pollock J. D. (2003). Making connections: the development of mesencephalic dopaminergic neurons. Brain Res. Dev. Brain Res. 147, 3–21. 10.1016/j.devbrainres.2003.09.010 14741747

[B249] Riegel A. C. Lupica C. R. (2004). Independent presynaptic and postsynaptic mechanisms regulate endocannabinoid signaling at multiple synapses in the ventral tegmental area. J. Neurosci. 24, 11070–11078. 10.1523/JNEUROSCI.3695-04.2004 15590923 PMC4857882

[B250] Robertson S. H. Rasmussen E. B. (2017). Effects of a cafeteria diet on delay discounting in adolescent and adult rats: alterations on dopaminergic sensitivity. J. Psychopharmacol. Oxf. Engl. 31, 1419–1429. 10.1177/0269881117735750 29067887

[B251] Rose J. E. Woolsey C. N. (1948). The orbitofrontal cortex and its connections with the mediodorsal nucleus in rabbit, sheep and cat. Res. Publ. Assoc. Res. Nerv. Ment. Dis. 27, 210–232. 18106857

[B252] Rosenberg D. R. Lewis D. A. (1994). Changes in the dopaminergic innervation of monkey prefrontal cortex during late postnatal development: a tyrosine hydroxylase immunohistochemical study. Biol. Psychiatry 36, 272–277. 10.1016/0006-3223(94)90610-6 7986893

[B253] Rubino T. Prini P. Piscitelli F. Zamberletti E. Trusel M. Melis M.. (2015). Adolescent exposure to THC in female rats disrupts developmental changes in the prefrontal cortex. Neurobiol. Dis. 73, 60–69. 10.1016/j.nbd.2014.09.015 25281318

[B254] Rubino T. Realini N. Braida D. Guidi S. Capurro V. Vigan,ò D.. (2009). Changes in hippocampal morphology and neuroplasticity induced by adolescent THC treatment are associated with cognitive impairment in adulthood. Hippocampus 19, 763–772. 10.1002/hipo.20554 19156848

[B255] Rubino T. Zamberletti E. Parolaro D. (2012). Adolescent exposure to cannabis as a risk factor for psychiatric disorders. J. Psychopharmacol. Oxf. Engl. 26, 177–188. 10.1177/0269881111405362 21768160

[B256] Salamone J. D. Correa M. (2012). The mysterious motivational functions of mesolimbic dopamine. Neuron 76, 470–485. 10.1016/j.neuron.2012.10.021 23141060 PMC4450094

[B257] Sallam N. A. Borgland S. L. (2021). Insulin and endocannabinoids in the mesolimbic system. J. Neuroendocrinol. 33, e12965. 10.1111/jne.12965 33856071

[B258] Sandoval-Salazar C. Ramírez-Emiliano J. Trejo-Bahena A. Oviedo-Solís C. I. Solís-Ortiz M. S. (2016). A high-fat diet decreases GABA concentration in the frontal cortex and hippocampus of rats. Biol. Res. 49, 15. 10.1186/s40659-016-0075-6 26927389 PMC4772645

[B259] Scherma M. Dess,ì C. Muntoni A. L. Lecca S. Satta V. Luchicchi A.. (2016). Adolescent Δ(9)-tetrahydrocannabinol exposure alters WIN55,212-2 self-administration in adult rats. Neuropsychopharmacol. Off. Publ. Am. Coll. Neuropsychopharmacol. 41, 1416–1426. 10.1038/npp.2015.295 26388146 PMC4793126

[B260] Schneider M. (2008). Puberty as a highly vulnerable developmental period for the consequences of cannabis exposure. Addict. Biol. 13, 253–263. 10.1111/j.1369-1600.2008.00110.x 18482434

[B261] Schneider M. (2013). Adolescence as a vulnerable period to alter rodent behavior. Cell Tissue Res. 354, 99–106. 10.1007/s00441-013-1581-2 23430475

[B262] Schneider M. Koch M. (2003). Chronic pubertal, but not adult chronic cannabinoid treatment impairs sensorimotor gating, recognition memory, and the performance in a progressive ratio task in adult rats. Neuropsychopharmacol. Off. Publ. Am. Coll. Neuropsychopharmacol. 28, 1760–1769. 10.1038/sj.npp.1300225 12888772

[B263] Schneider M. Schömig E. Leweke F. M. (2008). Acute and chronic cannabinoid treatment differentially affects recognition memory and social behavior in pubertal and adult rats. Addict. Biol. 13, 345–357. 10.1111/j.1369-1600.2008.00117.x 18782382

[B264] Schultz W. (2007). Multiple dopamine functions at different time courses. Annu. Rev. Neurosci. 30, 259–288. 10.1146/annurev.neuro.28.061604.135722 17600522

[B265] Seamans J. K. Yang C. R. (2004). The principal features and mechanisms of dopamine modulation in the prefrontal cortex. Prog. Neurobiol. 74, 1–58. 10.1016/j.pneurobio.2004.05.006 15381316

[B266] Sesack S. R. Hawrylak V. A. Matus C. Guido M. A. Levey A. I. (1998). Dopamine axon varicosities in the prelimbic division of the rat prefrontal cortex exhibit sparse immunoreactivity for the dopamine transporter. J. Neurosci. Off. J. Soc. Neurosci. 18, 2697–2708. 10.1523/JNEUROSCI.18-07-02697.1998 9502827 PMC6793120

[B267] Shansky R. M. Murphy A. Z. (2021). Considering sex as a biological variable will require a global shift in science culture. Nat. Neurosci. 24, 457–464. 10.1038/s41593-021-00806-8 33649507 PMC12900283

[B268] Simon N. W. Moghaddam B. (2014). Neural processing of reward in adolescent rodents. Dev. Cogn. Neurosci. 11, 145–154. 10.1016/j.dcn.2014.11.001 25524828 PMC4597598

[B269] Somerville L. H. Casey B. J. (2010). Developmental neurobiology of cognitive control and motivational systems. Curr. Opin. Neurobiol. 20, 236–241. 10.1016/j.conb.2010.01.006 20167473 PMC3014528

[B270] Sonntag K. C. Brenhouse H. C. Freund N. Thompson B. S. Puhl M. Andersen S. L. (2014). Viral over-expression of D1 dopamine receptors in the prefrontal cortex increase high-risk behaviors in adults: comparison with adolescents. Psychopharmacology 231, 1615–1626. 10.1007/s00213-013-3399-8 24408208 PMC3969417

[B271] South T. Huang X.-F. (2008). High-fat diet exposure increases dopamine D2 receptor and decreases dopamine transporter receptor binding density in the nucleus accumbens and caudate putamen of mice. Neurochem. Res. 33, 598–605. 10.1007/s11064-007-9483-x 17940894

[B272] Spear L. P. (2000). The adolescent brain and age-related behavioral manifestations. Neurosci. Biobehav. Rev. 24, 417–463. 10.1016/S0149-7634(00)00014-2 10817843

[B273] St Onge J. R. Ahn S. Phillips A. G. Floresco S. B. (2012). Dynamic fluctuations in dopamine efflux in the prefrontal cortex and nucleus accumbens during risk-based decision making. J. Neurosci. Off. J. Soc. Neurosci. 32, 16880–16891. 10.1523/JNEUROSCI.3807-12.2012 23175840 PMC6621758

[B274] Steele C. C. Pirkle J. R. A. Davis I. R. Kirkpatrick K. (2019). Dietary effects on the determinants of food choice: impulsive choice, discrimination, incentive motivation, preference, and liking in male rats. Appetite 136, 160–172. 10.1016/j.appet.2019.01.023 30721744 PMC6430664

[B275] Steinberg L. (2005). Cognitive and affective development in adolescence. Trends Cogn. Sci. 9, 69–74. 10.1016/j.tics.2004.12.005 15668099

[B276] Sturman D. A. Mandell D. R. Moghaddam B. (2010). Adolescents exhibit behavioral differences from adults during instrumental learning and extinction. Behav. Neurosci. 124, 16–25. 10.1037/a0018463 20141277 PMC2871391

[B277] Sturman D. A. Moghaddam B. (2011). The Neurobiology of Adolescence: changes in brain architecture, functional dynamics, and behavioral tendencies. Neurosci. Biobehav. Rev. 35, 1704–1712. 10.1016/j.neubiorev.2011.04.003 21527288 PMC3222328

[B278] Sturman D. A. Moghaddam B. (2012). Striatum processes reward differently in adolescents versus adults. Proc. Natl. Acad. Sci. U. S. A. 109, 1719–1724. 10.1073/pnas.1114137109 22307637 PMC3277117

[B279] Sugiura T. Kishimoto S. Oka S. Gokoh M. (2006). Biochemistry, pharmacology and physiology of 2-arachidonoylglycerol, an endogenous cannabinoid receptor ligand. Prog. Lipid Res. 45, 405–446. 10.1016/j.plipres.2006.03.003 16678907

[B280] Sugiura T. Kondo S. Sukagawa A. Nakane S. Shinoda A. Itoh K.. (1995). 2-Arachidonoylglycerol: a possible endogenous cannabinoid receptor ligand in brain. Biochem. Biophys. Res. Commun. 215, 89–97. 10.1006/bbrc.1995.2437 7575630

[B281] Surmeier D. J. Carrillo-Reid L. Bargas J. (2011). Dopaminergic modulation of striatal neurons, circuits and assemblies. Neuroscience 198, 3–18. 10.1016/j.neuroscience.2011.08.051 21906660 PMC3235731

[B282] Szabo B. Siemes S. Wallmichrath I. (2002). Inhibition of GABAergic neurotransmission in the ventral tegmental area by cannabinoids. Eur. J. Neurosci. 15, 2057–2061. 10.1046/j.1460-9568.2002.02041.x 12099913

[B283] Taber M. T. Das S. Fibiger H. C. (1995). Cortical regulation of subcortical dopamine release: mediation *via* the ventral tegmental area. J. Neurochem. 65, 1407–1410. 10.1046/j.1471-4159.1995.65031407.x 7643120

[B284] Tantot F. Parkes S. L. Marchand A. R. Boitard C. Naneix F. Layé S.. (2017). The effect of high-fat diet consumption on appetitive instrumental behavior in rats. Appetite 108, 203–211. 10.1016/j.appet.2016.10.001 27713085

[B285] Tarazi F. I. Baldessarini R. J. (2000). Comparative postnatal development of dopamine D(1), D(2) and D(4) receptors in rat forebrain. Int. J. Dev. Neurosci. Off. J. Int. Soc. Dev. Neurosci. 18, 29–37. 10.1016/S0736-5748(99)00108-2 10708903

[B286] Teegarden S. L. Scott A. N. Bale T. L. (2009). Early life exposure to a high fat diet promotes long-term changes in dietary preferences and central reward signaling. Neuroscience 162, 924–932. 10.1016/j.neuroscience.2009.05.029 19465087 PMC2723193

[B287] Tiklová K. Björklund Å. K. Lahti L. Fiorenzano A. Nolbrant S. Gillberg L.. (2019). Single-cell RNA sequencing reveals midbrain dopamine neuron diversity emerging during mouse brain development. Nat. Commun. 10, 581. 10.1038/s41467-019-08453-1 30718509 PMC6362095

[B288] Tirado-Muñoz J. Lopez-Rodriguez A. B. Fonseca F. Farré M. Torrens M. Viveros M.-P. (2020). Effects of cannabis exposure in the prenatal and adolescent periods: preclinical and clinical studies in both sexes. Front. Neuroendocrinol. 57, 100841. 10.1016/j.yfrne.2020.100841 32339546

[B289] Tsan L. Décarie-Spain L. Noble E. E. Kanoski S. E. (2021). Western diet consumption during development: setting the stage for neurocognitive dysfunction. Front. Neurosci. 15, 632312. 10.3389/fnins.2021.632312 33642988 PMC7902933

[B290] Tseng K.-Y. O'Donnell P. (2007). Dopamine modulation of prefrontal cortical interneurons changes during adolescence. Cereb. Cortex N. Y. N. 17, 1235–1240. 10.1093/cercor/bhl034 16818475 PMC2204087

[B291] Tseng K. Y. Mallet N. Toreson K. L. Le Moine C. Gonon F. O'Donnell P. (2006). Excitatory response of prefrontal cortical fast-spiking interneurons to ventral tegmental area stimulation *in vivo*. Synap. N. Y. N. 59, 412–417. 10.1002/syn.20255 16485264 PMC2190627

[B292] Tseng K. Y. O'Donnell P. (2005). Post-pubertal emergence of prefrontal cortical up states induced by D1-NMDA co-activation. Cereb. Cortex N. Y. N. 15, 49–57. 10.1093/cercor/bhh107 15217899

[B293] Turner K. M. Parkes S. L. (2020). Prefrontal regulation of behavioural control: evidence from learning theory and translational approaches in rodents. Neurosci. Biobehav. Rev. 118, 27–41. 10.1016/j.neubiorev.2020.07.010 32707346

[B294] Ueda N. Tsuboi K. (2012). Discrimination between two endocannabinoids. Chem. Biol. 19, 545–547. 10.1016/j.chembiol.2012.05.001 22633404

[B295] Ueda N. Tsuboi K. Uyama T. (2010). Enzymological studies on the biosynthesis of N-acylethanolamines. Biochim. Biophys. Acta 1801, 1274–1285. 10.1016/j.bbalip.2010.08.010 20736084

[B296] Urbonaite G. Knyzeliene A. Bunn F. S. Smalskys A. Neniskyte U. (2022). The impact of maternal high-fat diet on offspring neurodevelopment. Front. Neurosci. 16, 909762. 10.3389/fnins.2022.909762 35937892 PMC9354026

[B297] Urs N. M. Gee S. M. Pack T. F. McCorvy J. D. Evron T. Snyder J. C.. (2016). Distinct cortical and striatal actions of a β-arrestin-biased dopamine D2 receptor ligand reveal unique antipsychotic-like properties. Proc. Natl. Acad. Sci. U. S. A. 113, E8178–E8186. 10.1073/pnas.1614347113 27911814 PMC5167191

[B298] Uylings H. B. M. Groenewegen H. J. Kolb B. (2003). Do rats have a prefrontal cortex? Behav. Brain Res. 146, 3–17. 10.1016/j.bbr.2003.09.028 14643455

[B299] Van den Heuvel D. M. A. Pasterkamp R. J. (2008). Getting connected in the dopamine system. Prog. Neurobiol. 85, 75–93. 10.1016/j.pneurobio.2008.01.003 18304718

[B300] Van Eden C. G. Hoorneman E. M. D. Buijs R. M. Matthijssen M. A. H. Geffard M. Uylings H. B. M. (1987). Immunocytochemical localization of dopamine in the prefrontal cortex of the rat at the light and electron microscopical level. Neuroscience 22, 849–862. 10.1016/0306-4522(87)92964-2 3683852

[B301] Vander Weele C. M. Siciliano C. A. Matthews G. A. Namburi P. Izadmehr E. M. Espinel I. C.. (2018). Dopamine enhances signal-to-noise ratio in cortical-brainstem encoding of aversive stimuli. Nature 563, 397–401. 10.1038/s41586-018-0682-1 30405240 PMC6645392

[B302] Vendruscolo L. F. Gueye A. B. Darnaudéry M. Ahmed S. H. Cador M. (2010a). Sugar overconsumption during adolescence selectively alters motivation and reward function in adult rats. PLoS ONE 5, e9296. 10.1371/journal.pone.0009296 20174565 PMC2824808

[B303] Vendruscolo L. F. Gueye A. B. Vendruscolo J. C. M. Clemens K. J. Mormède P. Darnaudéry M.. (2010b). Reduced alcohol drinking in adult rats exposed to sucrose during adolescence. Neuropharmacology 59, 388–394. 10.1016/j.neuropharm.2010.05.015 20600175

[B304] Vertes R. P. (2004). Differential projections of the infralimbic and prelimbic cortex in the rat. Synap. N. Y. N. 51, 32–58. 10.1002/syn.10279 14579424

[B305] Volkow N. D. Wang G.-J. Baler R. D. (2011). Reward, dopamine and the control of food intake: implications for obesity. Trends Cogn. Sci. 15, 37–46. 10.1016/j.tics.2010.11.001 21109477 PMC3124340

[B306] Voorn P. Kalsbeek A. Jorritsma-Byham B. Groenewegen H. J. (1988). The pre- and postnatal development of the dopaminergic cell groups in the ventral mesencephalon and the dopaminergic innervation of the striatum of the rat. Neuroscience 25, 857–887. 10.1016/0306-4522(88)90041-3 3405431

[B307] Voorn P. Vanderschuren L. J. M. J. Groenewegen H. J. Robbins T. W. Pennartz C. M. A. (2004). Putting a spin on the dorsal-ventral divide of the striatum. Trends Neurosci. 27, 468–474. 10.1016/j.tins.2004.06.006 15271494

[B308] Walker D. M. Bell M. R. Flores C. Gulley J. M. Willing J. Paul M. J. (2017). Adolescence and reward: making sense of neural and behavioral changes amid the chaos. J. Neurosci. Off. J. Soc. Neurosci. 37, 10855–10866. 10.1523/JNEUROSCI.1834-17.2017 29118215 PMC5678018

[B309] Wang H. Treadway T. Covey D. P. Cheer J. F. Lupica C. R. (2015). Cocaine-induced endocannabinoid mobilization in the ventral tegmental area. Cell Rep. 12, 1997–2008. 10.1016/j.celrep.2015.08.041 26365195 PMC4857883

[B310] Wang Y. C. Bleich S. N. Gortmaker S. L. (2008). Increasing caloric contribution from sugar-sweetened beverages and 100% fruit juices among US children and adolescents, 1988–2004. Pediatrics 121, e1604–e1614. 10.1542/peds.2007-2834 18519465

[B311] Wickens J. R. Horvitz J. C. Costa R. M. Killcross S. (2007). Dopaminergic mechanisms in actions and habits. J. Neurosci. Off. J. Soc. Neurosci. 27, 8181–8183. 10.1523/JNEUROSCI.1671-07.2007 17670964 PMC6673057

[B312] Willing J. Cortes L. R. Brodsky J. M. Kim T. Juraska J. M. (2017). Innervation of the medial prefrontal cortex by tyrosine hydroxylase immunoreactive fibers during adolescence in male and female rats. Dev. Psychobiol. 59, 583–589. 10.1002/dev.21525 28561889 PMC5515298

[B313] Wilmouth C. E. Spear L. P. (2009). Hedonic sensitivity in adolescent and adult rats: taste reactivity and voluntary sucrose consumption. Pharmacol. Biochem. Behav. 92, 566–573. 10.1016/j.pbb.2009.02.009 19264093 PMC3385517

[B314] Wilson R. I. Nicoll R. A. (2001). Endogenous cannabinoids mediate retrograde signalling at hippocampal synapses. Nature 410, 588–592. 10.1038/35069076 11279497

[B315] Winstanley C. A. Eagle D. M. Robbins T. W. (2006). Behavioral models of impulsivity in relation to ADHD: translation between clinical and preclinical studies. Clin. Psychol. Rev. 26, 379–395. 10.1016/j.cpr.2006.01.001 16504359 PMC1892795

[B316] Wong A. Dogra V. R. Reichelt A. C. (2017). High-sucrose diets in male rats disrupt aspects of decision making tasks, motivation and spatial memory, but not impulsivity measured by operant delay-discounting. Behav. Brain Res. 327, 144–154. 10.1016/j.bbr.2017.03.029 28365197

[B317] Yee B. K. Keist R. von Boehmer L. Studer R. Benke D. Hagenbuch N.. (2005). A schizophrenia-related sensorimotor deficit links alpha 3-containing GABAA receptors to a dopamine hyperfunction. Proc. Natl. Acad. Sci. U. S. A. 102, 17154–17159. 10.1073/pnas.0508752102 16284244 PMC1288020

[B318] Yuan M. Cross S. J. Loughlin S. E. Leslie F. M. (2015). Nicotine and the adolescent brain. J. Physiol. 593, 3397–3412. 10.1113/JP270492 26018031 PMC4560573

[B319] Zachry J. E. Nolan S. O. Brady L. J. Kelly S. J. Siciliano C. A. Calipari E. S. (2021). Sex differences in dopamine release regulation in the striatum. Neuropsychopharmacol. Off. Publ. Am. Coll. Neuropsychopharmacol. 46, 491–499. 10.1038/s41386-020-00915-1 33318634 PMC8027008

[B320] Zhang H.-Y. Gao M. Liu Q.-R. Bi G.-H. Li X. Yang H.-J.. (2014). Cannabinoid CB2 receptors modulate midbrain dopamine neuronal activity and dopamine-related behavior in mice. Proc. Natl. Acad. Sci. U. S. A. 111, E5007–E5015. 10.1073/pnas.1413210111 25368177 PMC4246322

[B321] Zimmerman C. A. Knight Z. A. (2020). Layers of signals that regulate appetite. Curr. Opin. Neurobiol. 64, 79–88. 10.1016/j.conb.2020.03.007 32311645 PMC7572475

[B322] Zlebnik N. E. Cheer J. F. (2016). Drug-induced alterations of endocannabinoid-mediated plasticity in brain reward regions. J. Neurosci. 36, 10230–10238. 10.1523/JNEUROSCI.1712-16.2016 27707960 PMC5050322

[B323] Zuo X.-N. Kelly C. Di Martino A. Mennes M. Margulies D. S. Bangaru S.. (2010). Growing together and growing apart: regional and sex differences in the lifespan developmental trajectories of functional homotopy. J. Neurosci. 30, 15034–15043. 10.1523/JNEUROSCI.2612-10.2010 21068309 PMC2997358

